# Association of the triglyceride–glucose index with all-cause and cause-specific mortality: a population-based cohort study of 3.5 million adults in China

**DOI:** 10.1016/j.lanwpc.2024.101135

**Published:** 2024-07-06

**Authors:** Guangda He, Zenglei Zhang, Chunqi Wang, Wei Wang, Xueke Bai, Linkang He, Shi Chen, Guangyu Li, Yang Yang, Xiaoyan Zhang, Jianlan Cui, Wei Xu, Lijuan Song, Hao Yang, Wenyan He, Yan Zhang, Xi Li, Liang Chen

**Affiliations:** a National Clinical Research Center for Cardiovascular Diseases, State Key Laboratory of Cardiovascular Disease, Fuwai Hospital, National Center for Cardiovascular Diseases, Chinese Academy of Medical Sciences and Peking Union Medical College, Beijing, People’s Republic of China; b 4+4 Medical Doctor Program, Chinese Academy of Medical Sciences & Peking Union Medical College, Beijing, China

**Keywords:** Triglyceride-glucose index, Mortality, Cardiovascular disease, China, Cohort study

## Abstract

**Background:**

The triglyceride-glucose (TyG) index has been recognized as a crucial risk factor for cardiovascular diseases. However, the association between the TyG index and mortality in the general population remains elusive.

**Methods:**

Participants were enrolled from the China Health Evaluation And risk Reduction through nationwide Teamwork (ChinaHEART), a nationwide prospective cohort study. The outcomes of interest were all-cause, cardiovascular, and cancer mortality. Restricted cubic splines and Cox regression models were used to assess the associations between the TyG index and outcomes.

**Findings:**

In total, 3,524,459 participants with a median follow-up of 4.6 (IQR, 3.1–5.8) years were included. The associations of the TyG index with all-cause and cardiovascular mortality were reverse L-shaped, with cut-off values of 9.75 for all-cause mortality and 9.85 for cardiovascular mortality. For each 1-unit increase in the TyG index, when below the cut-off values, the TyG index was not significantly associated with all-cause mortality (HR = 1.02, 95% CI: 1.00–1.03) and was only modestly associated with cardiovascular mortality (HR = 1.09, 95% CI: 1.06–1.11). Conversely, when the cut-off values were exceeded, the HRs (95% CI) were 2.10 (1.94–2.29) for all-cause mortality and 1.99 (1.72–2.30) for cardiovascular mortality. However, the association between the TyG index and cancer mortality was linearly negative (HR = 0.97, 95% CI: 0.94–0.99).

**Interpretation:**

The associations of the TyG index with all-cause and cardiovascular mortality displayed reverse L-shaped patterns, while an elevated TyG index showed a slight negative association with cancer mortality. We suggest that <9.75 could be the optimal TyG index cut-off value among the Chinese general population. Individuals at high risk of mortality might benefit from proper management of a high TyG index.

**Funding:**

The National High Level Hospital Clinical Research Funding (2023-GSP-ZD-2, 2023-GSP-RC-01), the Ministry of Finance of China and National Health Commission of China.

**Research in context**

**Evidence before this study**

Insulin resistance (IR), characterized by decreased insulin sensitivity, hyperinsulinaemia, hyperglycaemia, endothelial dysfunction, systemic inflammation, and oxidative stress, is acknowledged as a crucial risk factor for cardiovascular diseases (CVDs). Due to the complexity of the direct measurement of IR, the triglyceride-glucose (TyG) index, a simple and reliable surrogate marker of IR, has been developed and widely used in clinical practice. The literature has revealed that the TyG index is strongly associated with increased mortality among patients with cardiovascular or metabolic diseases, including hypertension, diabetes, stroke, coronary heart disease, heart failure, and familial hypercholesterolemia. Nonetheless, there remains a scarcity of epidemiological and clinical evidence regarding the prognostic significance of the TyG index for all-cause and cause-specific mortality in the general population. The mere evidence of the association between the TyG index and mortality among the general population is mainly derived from American (the National Health and Nutrition Examination Survey, NHANES) and Korean study cohorts. Data are particularly scarce for other populations, especially people in low- and middle-income countries (LMICs), such as China.

**Added value of this study**

Based on a mega prospective cohort of 3.5 million participants from 31 provinces in the Chinese mainland, we evaluated the associations of the TyG index with all-cause, cardiovascular, and cancer mortality in the general population. Our findings revealed reverse L-shaped patterns in the associations of the TyG index with all-cause and cardiovascular mortality, and the cut-off values were 9.75 for all-cause death and 9.85 for cardiovascular death. Specifically, when the TyG index was below the cut-off values, it was not associated with all-cause mortality and was associated with only modestly greater (9%) cardiovascular mortality. In contrast, when over the cut-off values, a 1-unit increase in the TyG index was associated with 110% and 99% greater risks of all-cause and cardiovascular deaths, respectively. However, the association between the TyG index and cancer was linear, and a higher TyG index was associated with slightly lower (3%) cancer mortality. The associations were consistent in the subgroups stratified by arteriosclerotic cardiovascular disease risk, sex, age, central obesity status, medical history of hypertension and diabetes, and antidiabetic drug use.

**Implications of all the available evidence**

To the best of our knowledge, this is the largest study to evaluate the associations of the TyG index with all-cause and cause-specific mortality. Our findings provided novel insight that the TyG index could be a simple and practical tool for screening people at high risk of mortality, and all people with TyG index values greater than 9.75 are vulnerable to all-cause mortality and should receive targeted attention. Furthermore, given the simplicity of the measurement and calculation of the TyG index, its implementation could prove feasible even in limited-resource settings, particularly in LMICs.

## Introduction

With economic development, lifestyle changes, and accelerated population ageing in recent decades, cardiovascular disease (CVD) has become a major health burden globally, affecting 621 million people and contributing to 20.5 million deaths annually.^1^ Insulin resistance (IR), a pathophysiological process characterized by impaired signal transduction and biological actions in response to insulin stimulation, impaired insulin sensitivity of target tissues or organs, and hyperinsulinaemia, plays important roles in the initiation and progression of CVDs.^2^, ^3^, ^4^, ^5^, ^6^ Although the hyperinsulinaemic–euglycaemic clamp has been considered the gold standard for assessing IR, it is too expensive and complex to be widely applied in the clinic.^7^ Fortunately, in recent years, the triglyceride-glucose (TyG) index, a simple and effective surrogate marker of IR, has been developed and shown to have high sensitivity for detecting IR.^8^^,^^9^ People with elevated TyG index levels are vulnerable to abnormal glucose and lipid metabolism, endothelial dysfunction, coagulation disorders, and smooth muscle cell dysfunction, all of which contribute to the pathogenesis of CVDs.^9^ Therefore, the TyG index has been recognized as a remarkable metabolic risk factor for cardiovascular events among patients with cardiovascular or metabolic diseases.^9^, ^10^, ^11^, ^12^, ^13^

However, in the general population, the associations between the TyG index and mortality remain inconsistent across previous studies.^14^ For instance, in a regional Chinese population-based cohort, Cai et al. reported J-shaped associations of the TyG index with all-cause and cardiovascular death.^15^ Nevertheless, a series of analyses based on the National Health and Nutrition Examination Survey (NHANES) cohort revealed U-shaped associations between the TyG index and mortality.^16^, ^17^, ^18^ Moreover, a few studies demonstrated sex differences. Specifically, Yu et al. identified a U-shaped association between the TyG index and cardiovascular mortality in males but an L-shaped association in females.^17^ In a large-scale Korean cohort, Kim et al. observed an association between the TyG index and all-cause death in females only, and there was no significant association between the TyG index and cardiovascular death in either sex.^19^ In addition, an analysis of the Prospective Urban Rural Epidemiology (PURE) study revealed that a higher TyG index was associated with excess cardiovascular mortality only in low-income countries and not in middle- or high-income countries.^20^ Nevertheless, prior studies exploring the association between the TyG index and mortality have been conducted mainly in developed countries, such as the United States and Korea.^16^, ^17^, ^18^, ^19^^,^^21^, ^22^, ^23^, ^24^, ^25^, ^26^ Clinical and epidemiological evidence regarding whether the TyG index is associated with mortality among the general population in low- and middle-income countries (LMICs) is scant. Therefore, to thoroughly comprehend the prognostic implications of the TyG index and provide a practical metabolic tool for assessing death risks in the general population, a large-scale prospective study based on the general population in an LMIC is needed to evaluate the association of the TyG index with all-cause and cause-specific mortality.

To address the aforementioned knowledge gaps, we conducted analyses utilizing data from a national cohort study covering all 31 provinces in the Chinese mainland and explored the associations of the TyG index with all-cause and cause-specific mortality in the general population.

## Methods

### Study design and participants

The China Health Evaluation And risk Reduction through nationwide Teamwork (ChinaHEART, formerly named China-PEACE [Patient-Centered Evaluative Assessment of Cardiac Events] Million Persons Project [MPP]) is a nationwide population-based screening project, and the detailed study design has been published previously.^27^, ^28^, ^29^ In brief, based on the primary health care institution system in China (community health stations, community health centres, township health centres, and village clinics), study sites were selected to reflect the diversity in population structure, geographical distribution, and economic development of the country, and 353 sites (212 rural, 141 urban) across all 31 provinces in the Chinese mainland were selected. At each site, participants were invited and recruited via extensive publicity campaigns in the newspapers and on television. Eligible participants were local residents in the selected region aged 35–75 years. Specifically, participants were defined as local residents if they lived at the study site for ≥6 months within 1 year before the baseline interview date. In total, 4,404,586 participants were recruited from 2014 to 2022, and those without fasting glucose or lipid data were excluded (n = 880,127) from the current analyses.

This ChinaHEART project adhered to the Declaration of Helsinki and was approved by the central ethics committee at Fuwai Hospital (2014-574), and all recruited participants provided signed informed consent. The study was registered at ClinicalTrials.gov (NCT02536456).

### Data collection, definitions, and covariates

Data on anthropometrics (weight, height, and waist circumference), blood pressure (BP), fasting glucose concentrations, fasting lipid concentrations, socioeconomic status (marital status, education, annual household income, medical insurance, and residence [urban or rural]), region (north or south), lifestyle risk factors (current smoker or drinker), self-reported medical history (hypertension, diabetes, chronic obstructive pulmonary disease [COPD], and cancer), and medications (use of antihypertensive, antidiabetic, and lipid-lowering drugs) were collected at baseline interviews. Central obesity was defined as a waist circumference ≥90 cm in men and ≥85 cm in women.^30^^,^^31^ BP was measured twice on the right upper arm after 5 min of rest in a seated position, with at least a 1-min delay between the 2 measurements. If the difference between the 2 measurements exceeded 10 mmHg, a third measurement was performed, and the mean value of the last 2 measurements was recorded as the BP level. Fasting blood glucose was assayed using a rapid blood glucose analyser. Total cholesterol (TC), triglycerides (TG), and high-density lipoprotein cholesterol (HDL-C) concentrations were measured using a rapid lipid analyser, and low-density lipoprotein cholesterol (LDL-C) concentrations were calculated with the Friedewald equation.^32^ The TyG index was calculated using ln[fasting blood TG (mg/dl) × fasting blood glucose (mg/dl)/2].^8^ Participants were defined as current drinkers if they consumed alcohol ≥2 times per week in the last 12 months. Hypertension status was defined according to participants’ self-reported medical history or use of antihypertensive medication(s). Diabetes was defined as a fasting glucose level ≥126 mg/dL (7 mmol/L), self-reported history of diabetes, or use of antidiabetic medication(s).

Moreover, participants were stratified by their arteriosclerotic cardiovascular disease (ASCVD) risk^33^: 1) secondary prevention group: established ASCVD (i.e., history of coronary heart disease, peripheral arterial disease, or ischaemic stroke); 2) primary prevention group: high ASCVD risk (predicted 10-year risk for ASCVD ≥10% according to the China-PAR risk algorithm) without established ASCVD; and 3) low-risk group: without high estimated CVD risk (predicted 10-year risk for ASCVD <10% according to the China-PAR risk algorithm) or established ASCVD.

### Study outcomes

Death data were obtained by linking the National Mortality Surveillance System and Vital Registration of the Chinese Center for Disease Control and Prevention database. Death data were collected until December 31, 2022.^34^ The death data in the database were reported by health care institutions, and the data were rechecked against local residential records and health insurance records annually. In the current analyses, the primary outcome was all-cause death, and the secondary outcomes were cardiovascular death (ICD-10: I00–I99) and cancer death (ICD-10: C00–C99).

### Statistical analysis

The median (interquartile range, IQR) was used to present continuous variables, and the frequency (percent) was used for categorical variables.

First, participants were classified into 4 groups by their TyG index quartiles (quartile 1: TyG index <8.41; quartile 2: 8.41 ≤ TyG index <8.76; quartile 3: 8.76 ≤ TyG index <9.16; quartile 4: TyG index ≥9.16). Kaplan–Meier curves and log-rank tests were used to illustrate and compare the cumulative outcome incidences across participant groups. Multivariable Cox proportional hazard models were used to estimate the hazard ratios (HRs) and 95% confidence intervals (95% CIs) between the TyG index quartiles and outcomes. The proportional hazard assumption was checked by Schoenfeld residual graphs, and no violation was observed. Covariates were selected based on a literature review and clinical experience, including age, sex (male or female), education (with or without a college degree), annual household income (<50,000 RMB or ≥ 50,000 RMB), current smoking status (yes or no), current alcohol consumption status (yes or no), central obesity status (yes or no), systolic BP, LDL-C concentrations, HDL-C concentrations, medical history (COPD, cancer, diabetes), and use of lipid-lowering drugs (yes or no). Noncardiovascular and noncancer deaths were defined as competing risks when analysing the associations of the TyG index with cardiovascular and cancer mortality, respectively. Second, we used the TyG index as a continuous variable and re-evaluated its associations with outcomes. Restricted cubic splines (RCSs) were employed to investigate the dose‒response relationship between the TyG index and outcomes, using the lowest risk as the reference. Based on a literature review, we tried 3–5 knots to fit the RCSs and used the Akaike information criterion (AIC) to determine the knots. According to the minimum AIC, we finally used 3 knots to examine the dose‒response associations. If the association was nonlinear and approximated a U-, L-, or reverse L-shaped pattern, a two-line piecewise linear model with a single change point was applied to explore the change point (cut-off value) of the TyG index with the highest likelihood.^35^ Furthermore, the associations of the TyG index with outcomes below and above the cut-off value were re-examined using multivariable Cox proportional hazard models. To evaluate the robustness of whether the associations were modified by covariates, we further performed subgroup analyses of participants' ASCVD risk status (low-risk, primary prevention, secondary prevention), sex (male/female), age (quartile 1 to quartile 4), central obesity status (yes, no), LDL-C concentrations (LDL-C < 55 mg/dL, 55 mg/dL ≤ LDL-C < 70 mg/dL, 70 mg/dL ≤ LDL-C < 100 mg/dL, LDL-C ≥ 100 mg/dL), hypertension status (yes/no), diabetes status (yes/no), and antidiabetic drug use status.

To test the robustness of our findings, we performed 2 sensitivity analyses: i) adjustment for non-HDL-C concentrations instead of LDL-C concentrations in the statistical models and ii) re-examination of the associations among participants not taking lipid-lowering drugs.

The rates of missing data were ≤0.22% (missing BMI data: n = 7735; missing systolic BP data: n = 109; missing waist circumference data: n = 1736), and missing data were imputed using the mean values. A 2-sided P < 0.05 was considered to indicate statistical significance. All analyses were performed with R software version 4.3.2 (R Foundation for Statistical Computing, Vienna, Austria) and SAS software (version 9.4, SAS Institute, Cary, NC).

### Role of the funding source

The funding body had no role in the study design, data collection, statistical analysis, manuscript writing, or data interpretation for the current study.

## Results

### Participant characteristics

In total, 3,524,459 subjects from the ChinaHEART cohort were included in this study. The characteristics of the included and excluded participants are shown in Supplement Table S1, and the included participants were more likely to be female. Among the included participants, the median age was 56 years (IQR: 49–64), and 60.6% of the participants were female. The median TyG index, TG concentration, and glucose concentration were 8.76 (IQR: 8.41–9.16), 120 (IQR: 88–170) mg/dL, and 104 (IQR: 95–117) mg/dL, respectively (Table 1). Participant characteristics according to the TyG index quartiles are summarized in Table 1. Individuals with higher quartiles of the TyG index were older and had higher BP, BMI, waist circumference, TC concentrations, TG concentrations, and glucose concentrations. They were also more likely to reside in urban areas and the northern region, be complicated with comorbidities (central obesity, hypertension, and diabetes), take lipid-lowering and antidiabetic drugs, and be classified into primary and secondary ASCVD prevention groups.

**Table 1:** Participant characteristics by the triglyceride-glucose index quartiles.

Characteristic^a^	Overall (n = 3,524,459)	Quartile 1 (n = 880,296)	Quartile 2 (n = 883,137)	Quartile 3 (n = 879,621)	Quartile 4 (n = 881,405)
**Age, year**	56 (49, 64)	55 (47, 64)	56 (48, 64)	57 (49, 64)	57 (50, 64)
**Female**	2,136,709 (60.6)	511,695 (58.1)	537,433 (60.9)	545,851 (62.1)	541,730 (61.5)
**Clinical characteristics**					
SBP, mmHg	134 (122, 148)	129 (118, 143)	132 (121, 145)	135 (123, 149)	139 (127, 153)
DBP, mmHg	81 (74, 88)	79 (72, 86)	80 (73, 87)	81 (75, 89)	83 (76, 91)
Body mass index, kg/m^3^	24.5 (22.4, 26.9)	23.3 (21.4, 25.4)	24.2 (22.2, 26.4)	24.9 (22.9, 27.2)	25.7 (23.7, 28.0)
Waist, cm	84 (78, 90)	80 (74, 86)	83 (77, 89)	85 (79, 91)	87 (81, 93)
Central obesity	1,349,181 (38.3)	210,776 (23.9)	297,410 (33.7)	374,316 (42.6)	466,679 (52.9)
**Marital status**					
Married	3,276,299 (93.0)	821,941 (93.4)	821,203 (93.0)	816,458 (92.8)	816,697 (92.7)
Unmarried	211,593 (6.0)	49,163 (5.6)	52,549 (6.0)	53,724 (6.1)	56,157 (6.4)
Unknown	36,567 (1.0)	9192 (1.0)	9385 (1.1)	9439 (1.1)	8551 (1.0)
**Education**					
Primary school or lower	1,576,464 (44.7)	404,311 (45.9)	396,781 (44.9)	389,938 (44.3)	385,434 (43.7)
Middle school	1,130,784 (32.1)	280,023 (31.8)	282,355 (32.0)	282,564 (32.1)	285,842 (32.4)
High school	504,474 (14.3)	117,110 (13.3)	125,024 (14.2)	129,356 (14.7)	132,984 (15.1)
College or higher	273,881 (7.8)	68,708 (7.8)	68,709 (7.8)	67,930 (7.7)	68,534 (7.8)
Unknown	38,856 (1.1)	10,144 (1.2)	10,268 (1.2)	9833 (1.1)	8611 (1.0)
**Last year household income**					
<10,000 RMB	605,346 (17.2)	156,814 (17.8)	154,754 (17.5)	148,956 (16.9)	144,822 (16.4)
10,000 ≤ income <50,000 RMB	1,932,326 (54.8)	481,805 (54.7)	481,248 (54.5)	481,352 (54.7)	487,921 (55.4)
≥50,000 RMB	659,593 (18.7)	158,902 (18.1)	163,870 (18.6)	167,923 (19.1)	168,898 (19.2)
Unknown	327,194 (9.3)	82,775 (9.4)	83,265 (9.4)	81,390 (9.3)	79,764 (9.0)
**Medical insurance**					
With medical insurance	3,443,302 (97.7)	859,465 (97.6)	861,556 (97.6)	859,416 (97.7)	862,865 (97.9)
Without medical insurance	11,851 (0.3)	3749 (0.4)	3030 (0.3)	2603 (0.3)	2469 (0.3)
Unknown	69,306 (2.0)	17,082 (1.9)	18,551 (2.1)	17,602 (2.0)	16,071 (1.8)
**Residence**					
Urban	1,404,386 (39.8)	336,259 (38.2)	350,077 (39.6)	356,904 (40.6)	361,146 (41.0)
Rural	2,120,073 (60.2)	544,037 (61.8)	533,060 (60.4)	522,717 (59.4)	520,259 (59.0)
**Region**					
North	1,420,949 (40.3)	310,649 (35.3)	351,040 (39.7)	373,880 (42.5)	385,380 (43.7)
South	2,103,510 (59.7)	569,647 (64.7)	532,097 (60.3)	505,741 (57.5)	496,025 (56.3)
**Current smoker**	677,469 (19.2)	178,244 (20.2)	163,502 (18.5)	160,573 (18.3)	175,150 (19.9)
**Current drinker**	814,161 (23.1)	211,231 (24.0)	195,631 (22.2)	194,342 (22.1)	212,957 (24.2)
**Medical history**					
Hypertension	1,435,019 (40.7)	281,182 (31.9)	327,407 (37.1)	374,242 (42.5)	452,188 (51.3)
Diabetes	254,353 (7.2)	14,343 (1.6)	31,448 (3.6)	58,362 (6.6)	150,200 (17.0)
COPD	8331 (0.2)	2562 (0.3)	2226 (0.3)	1890 (0.2)	1653 (0.2)
Cancer	14,033 (0.4)	3288 (0.4)	3368 (0.4)	3545 (0.4)	3832 (0.4)
**Fasting lipid, mg/dL**					
TC	173 (148, 200)	161 (139, 186)	169 (147, 195)	177 (153, 203)	188 (161, 217)
LDL-C	92 (71, 115)	85 (67, 106)	92 (73, 114)	96 (75, 119)	94 (70, 120)
TG	120 (88, 170)	74 (65, 84)	105 (94, 117)	144 (127, 161)	220 (183, 275)
HDL-C	53 (44, 65)	59 (49, 71)	55 (46, 66)	52 (43, 62)	48 (40, 57)
**Fasting glucose, mg/dL**	104 (95, 117)	95 (88, 104)	103 (94, 112)	106 (97, 119)	117 (103, 140)
**TyG index**	8.76 (8.41, 9.16)	8.21 (8.06, 8.32)	8.59 (8.50, 8.67)	8.94 (8.85, 9.04)	9.46 (9.29, 9.71)
**Using lipid-lowering drugs**	100,549 (2.9)	14,386 (1.6)	20,239 (2.3)	26,653 (3.0)	39,271 (4.5)
**Using lipid antidiabetic drugs**	223,213 (6.3)	13,475 (1.5)	28,069 (3.2)	51,326 (5.8)	130,343 (14.8)
**ASCVD risk group**					
Low-risk	2,642,733 (75.0)	744,891 (84.6)	701,606 (79.4)	648,316 (73.7)	547,920 (62.2)
Primary prevention	771,712 (21.9)	115,453 (13.1)	156,724 (17.8)	201,614 (22.9)	297,921 (33.8)
Secondary prevention	110,014 (3.1)	19,952 (2.3)	24,807 (2.8)	29,691 (3.4)	35,564 (4.0)

Abbreviations: TyG index: triglyceride-glucose index; SBP: systolic blood pressure; DBP: diastolic blood pressure; COPD: chronic obstructive pulmonary disease; TC: total cholesterol; LDL-C: low-density lipoprotein cholesterol; TG: triglyceride; HDL-C: high-density lipoprotein cholesterol; ASCVD: arteriosclerotic cardiovascular disease.

a Results are presented as interquartile range for continuous variables or number (percentage) for categorical variables.

### Associations between the triglyceride-glucose index and the risk of all-cause, cardiovascular, and cancer mortality

**Fig. 1:**
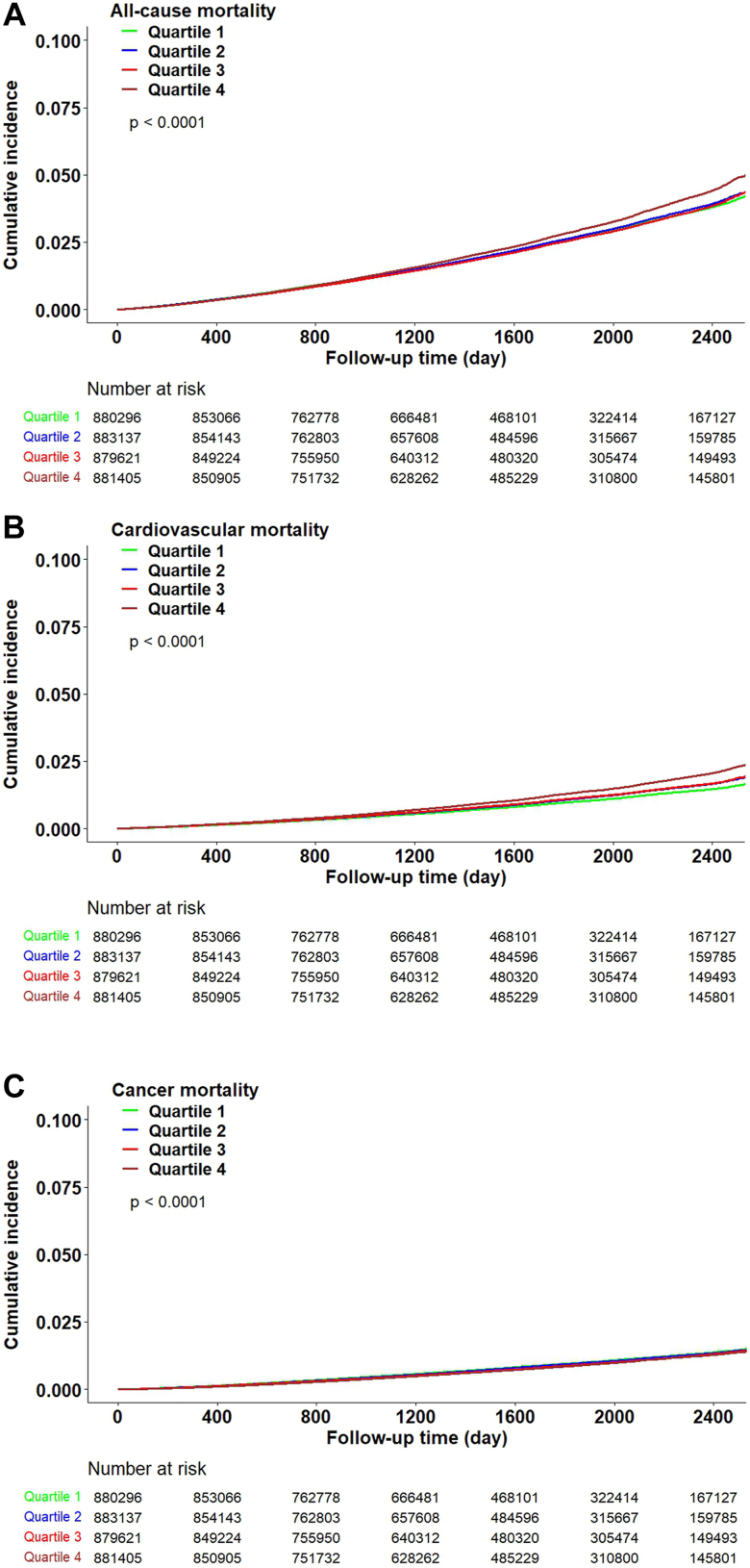
**Unadjusted Kaplan–Meier curves**. A) All-cause mortality by the triglyceride-glucose index quartiles; B) Cardiovascular mortality by the triglyceride-glucose index quartiles; C) Cancer mortality by the triglyceride-glucose index quartiles.

**Fig. 2:**
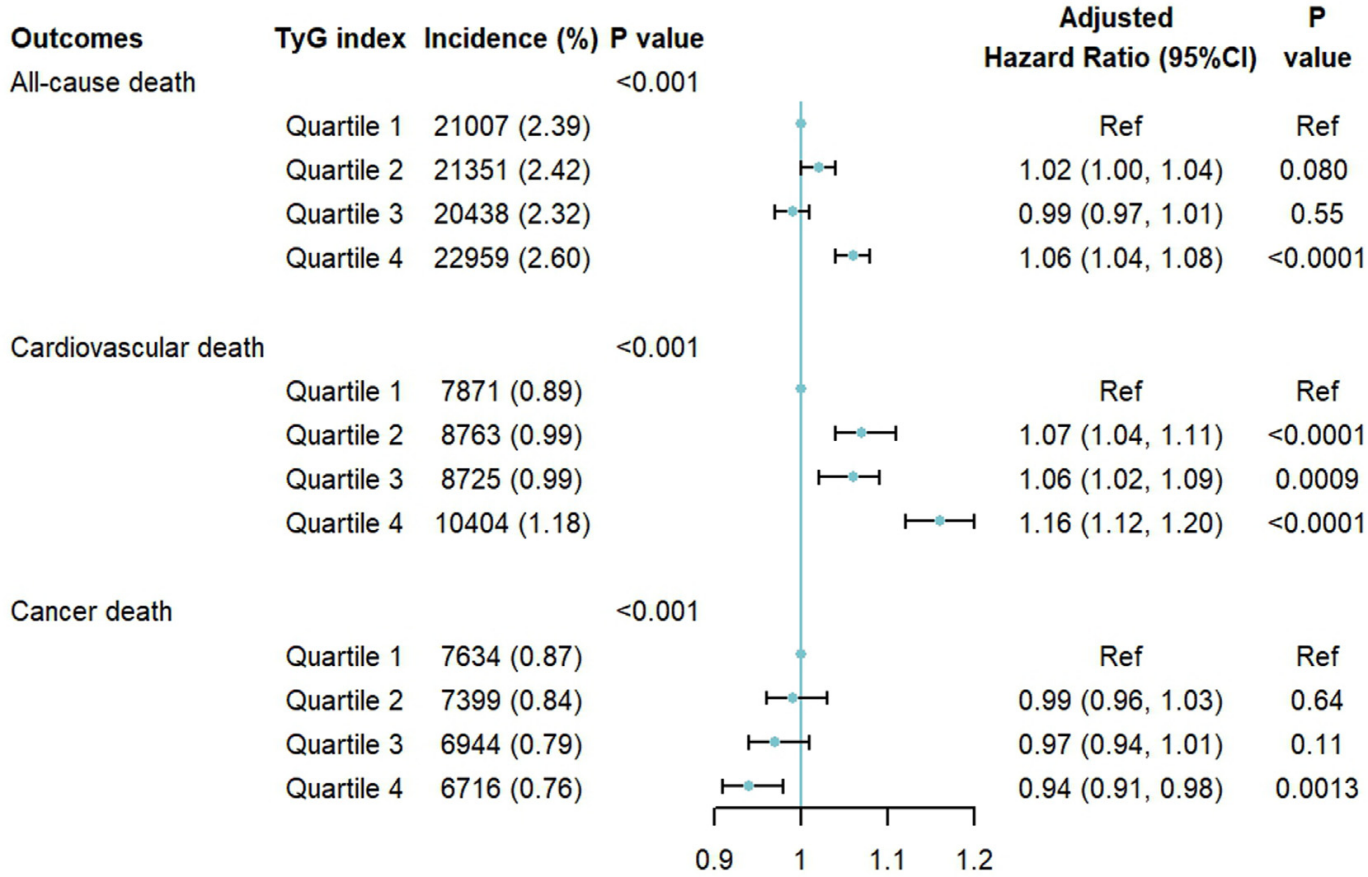
**Adjusted associations of the triglyceride-glucose index quartiles with all-cause, cardiovascular, and cancer mortality**. Notes: Adjust for: age, sex (male or female), education (with or without a college degree), annual household income (<50,000 RMB or ≥50,000 RMB), current smoker (yes or no), current drinker (yes or no), central obesity (yes or no), systolic blood pressure, low-density lipoprotein cholesterol, high-density lipoprotein cholesterol, medical history (chronic obstructive pulmonary disease, cancer, diabetes), and using lipid-lowering drug (yes or no).

During a median follow-up of 4.6 (IQR: 3.1–5.8) years, 85,755 (2.4%) participants died, among whom 35,763 (41.7%) died from cardiovascular causes and 28,693 (33.5%) died from cancer. The Kaplan–Meier curves revealed that individuals with a TyG index in quartile 4 had significantly greater incidences of all-cause death (2.60%) and cardiovascular death (1.18%) than did those with a TyG index in the other quartiles; however, the incidence of cancer death was relatively lower for individuals with a TyG index in quartile 4 (Figs. 1 and 2).

The adjusted associations between the TyG index and outcomes are shown in Fig. 2. Compared with the reference group (quartile 1), quartile 4 had significantly greater all-cause mortality (quartile 2: HR 1.02, 95% CI: 1.00–1.04; quartile 3: HR 0.99, 95% CI: 0.97–1.01; quartile 4: HR 1.06, 95% CI: 1.04–1.08). Moreover, cardiovascular mortality was significantly greater in the quartile 2 (HR 1.07, 95% CI: 1.04–1.11), quartile 3 (HR 1.06, 95% CI: 1.02–1.09), and quartile 4 (HR 1.16, 95% CI: 1.12–1.20) groups. However, as the TyG index increased, cancer mortality decreased (quartile 2: HR 0.99, 95% CI: 0.96–1.03; quartile 3: HR 0.97, 95% CI: 0.94–1.01; quartile 4: HR 0.94, 95% CI: 0.91–0.98).

**Fig. 3:**
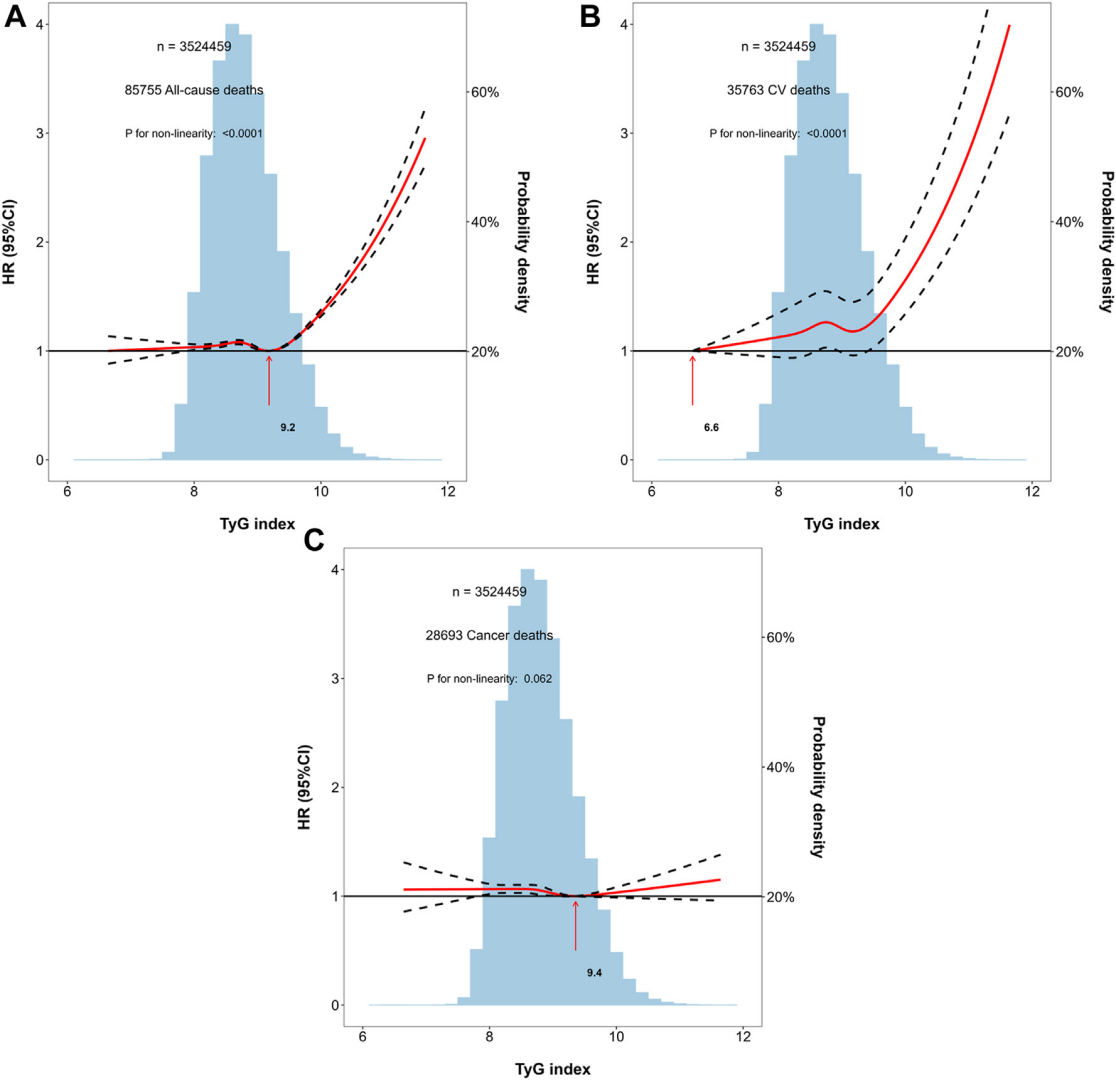
**Adjusted dose–respond associations of the triglyceride-glucose index with outcomes**. A) Adjusted dose–respond association of the triglyceride-glucose index with all-cause mortality; B) Adjusted dose–respond association of the triglyceride-glucose index with cardiovascular mortality; C) Adjusted dose–respond association of the triglyceride-glucose index with cancer mortality. Abbreviation: HR: hazard ratio; CI: confidence interval; TyG index: triglyceride-glucose index; CV: cardiovascular. Notes: Adjust for: age, sex (male or female), education (with or without a college degree), annual household income (<50,000 RMB or ≥50,000 RMB), current smoker (yes or no), current drinker (yes or no), central obesity (yes or no), systolic blood pressure, low-density lipoprotein cholesterol, high-density lipoprotein cholesterol, medical history (chronic obstructive pulmonary disease, cancer, diabetes), and using lipid-lowering drug (yes or no).

The adjusted dose‒response associations between the TyG index and outcomes are shown in Fig. 3. The associations of the TyG index with all-cause and cardiovascular mortality were nonlinear (P < 0.0001) and displayed reverse L-shaped patterns, while the association between the TyG index and cancer mortality was linear (P = 0.062). Using two-line piecewise linear models, the estimated cut-off values are shown in Table 2. The estimated cut-off values for all-cause and cardiovascular mortality in the overall sample were 9.75 (95% CI: 9.72, 9.78) and 9.85 (95% CI: 9.82, 9.88), respectively.

**Table 2:** Estimated cut-off values in the associations of the triglyceride-glucose index with all-cause and cardiovascular mortality.

Participants	Triglyceride-glucose index change point (95% CI)	
	All-cause mortality	Cardiovascular mortality
Overall	9.75 (9.72, 9.78)	9.85 (9.82, 9.88)
Overall for sensitivity 1∗	9.79 (9.76, 9.82)	9.87 (9.84, 9.90)
Overall for sensitivity 2^#^	9.80 (9.77, 9.83)	9.88 (9.85, 9.91)
Low-risk	9.73 (9.69, 9.76)	9.86 (9.82, 9.89)
Primary prevention	9.89 (9.87, 9.92)	9.94 (9.91, 9.96)
Secondary prevention	9.69 (9.67, 9.72)	9.67 (9.64, 9.70)
Male	9.68 (9.66, 9.71)	9.80 (9.77, 9.83)
Female	9.72 (9.69, 9.75)	9.72 (9.68, 9.75)
Age quartile 1	9.65 (9.62, 9.67)	9.78 (9.74, 9.81)
Age quartile 2	9.66 (9.63, 9.69)	9.79 (9.76, 9.82)
Age quartile 3	9.72 (9.69, 9.74)	9.76 (9.74, 9.79)
Age quartile 4	9.74 (9.71, 9.77)	9.82 (9.79, 9.85)
Non central obesity	9.68 (9.65, 9.71)	9.73 (9.70, 9.76)
Central obesity	9.75 (9.72, 9.77)	9.84 (9.81, 9.87)
LDL-C < 55 mg/dL	9.79 (9.74, 9.83)	9.88 (9.83, 9.92)
55 ≤ LDL-C < 70 mg/dL	9.66 (9.63, 9.69)	9.80 (9.75, 9.85)
70 ≤ LDL-C < 100 mg/dL	9.73 (9.69, 9.76)	9.83 (9.79, 9.86)
LDL-C ≥ 100 mg/dL	9.85 (9.82, 9.88)	9.89 (9.86, 9.92)
Non-hypertension	9.70 (9.67, 9.72)	9.79 (9.76, 9.82)
Hypertension	9.76 (9.74, 9.79)	9.84 (9.81, 9.86)
Non-diabetes	9.66 (9.63, 9.68)	9.76 (9.73, 9.80)
Diabetes	10.01 (9.98, 10.04)	9.79 (9.75, 9.83)
Diabetes without antidiabetic drug	9.57 (9.54, 9.60)	9.43 (9.39, 9.47)
Diabetes with antidiabetic drug	10.03 (9.99, 10.06)	9.81 (9.77, 9.85)

Abbreviation: CI: confidence interval; LDL-C: low-density lipoprotein cholesterol.

Notes: sensitivity 1∗: adjust for non-high-density lipoprotein cholesterol instead of low-density lipoprotein cholesterol; sensitivity 2^#^: participants without using lipid-lowering drugs.

**Fig. 4:**
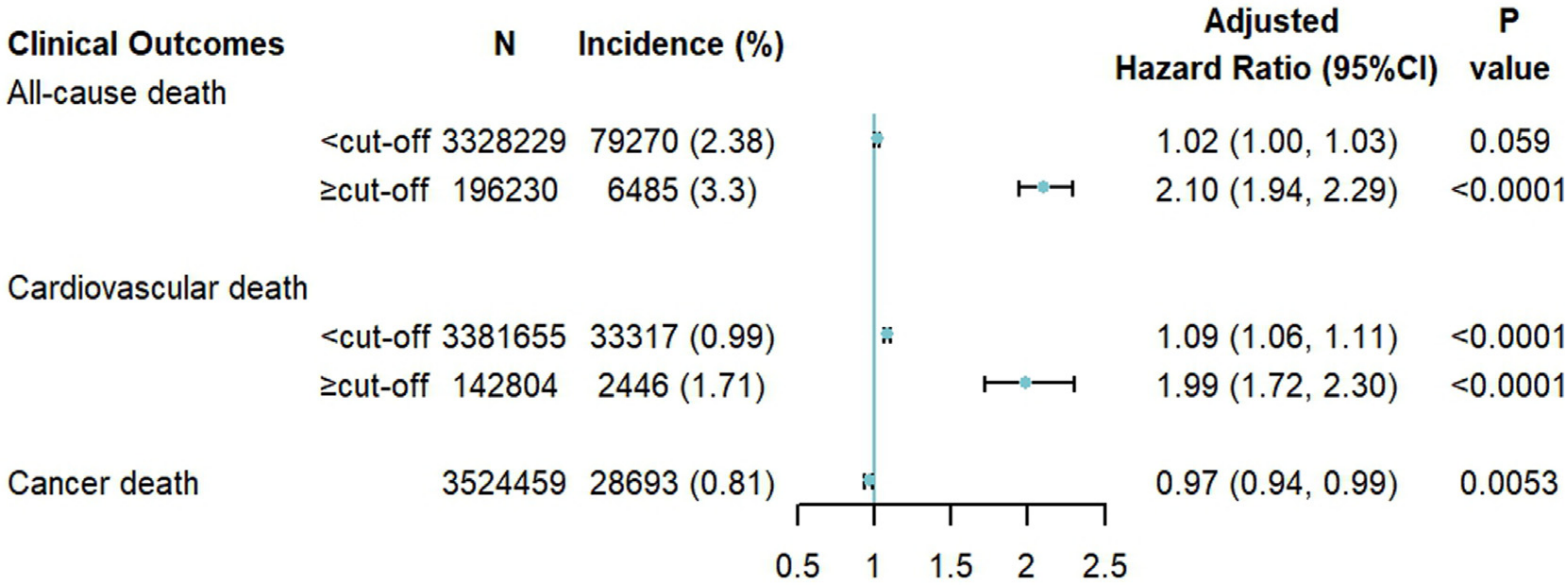
**Adjusted associations of the triglyceride-glucose index with all-cause, cardiovascular, and cancer mortality**. Notes: Adjust for: age, sex (male or female), education (with or without a college degree), annual household income (<50,000 RMB or ≥ 50,000 RMB), current smoker (yes or no), current drinker (yes or no), central obesity (yes or no), systolic blood pressure, low-density lipoprotein cholesterol, high-density lipoprotein cholesterol, medical history (chronic obstructive pulmonary disease, cancer, diabetes), and using lipid-lowering drug (yes or no).

Based on the estimated cut-off values, we re-examined the associations of the TyG index as a continuous variable with all-cause, cardiovascular, and cancer mortality (Fig. 4). Below the cut-off values, the TyG index was not significantly associated with all-cause mortality (HR 1.02, 95% CI: 1.00–1.03) and was only slightly related to greater cardiovascular mortality (HR 1.09, 95% CI: 1.06–1.11). However, when above the cut-off values, a 1-unit increase in the TyG index was associated with 110% greater all-cause mortality (HR 2.10, 95% CI: 1.94–2.29) and 99% greater cardiovascular mortality (HR 1.99, 95% CI: 1.72–2.30). Additionally, considering that the association between the TyG index and cancer mortality was linear, we evaluated this association and found that increasing the TyG index was associated with slightly lower cancer mortality (HR 0.97, 95% CI: 0.94–0.99).

### Subgroup analyses

The dose–response associations of the TyG index with all-cause, cardiovascular, and cancer mortality are illustrated in Supplement Figures S1–S8, and the trends were similar across various subgroups. The estimated cut-off values for the subgroup analyses are shown in Table 2, and all the cut-off values ranged from 9.43 to 10.03. Similarly, a higher TyG index was associated with substantially increased risks of all-cause and cardiovascular death when above the cut-off values but not below the cut-off values (Supplement Figures S1–S8).

### Sensitivity analyses

In the sensitivity analyses, the results were largely unchanged when adjusting for non-HDL-C instead of LDL-C (Supplement Figure S9). Similar results were observed when individuals taking lipid-lowering drugs were excluded (Supplement Figure S10).

## Discussion

In this mega prospective cohort study covering 31 provinces across the Chinese mainland, we investigated the associations between the TyG index and all-cause and cause-specific mortality in the general population. We observed reverse L-shaped associations between the TyG index and the risk of all-cause and cardiovascular death. Specifically, the cut-off values were 9.75 and 9.85 for all-cause and cardiovascular mortality, respectively. An elevated TyG index was significantly associated with substantially increased all-cause and cardiovascular mortality when the TyG index was above the respective cut-off value. Moreover, we observed that an increasing TyG index was associated with slightly lower cancer mortality. Our findings suggest that the TyG index, a simple and validated surrogate biomarker of IR, could be used as an effective tool for screening people at high risk of all-cause and cardiovascular death in the general population.

We performed a short systematic review in Supplemental Material Part 1 and found that the prognostic value of the TyG index for mortality was inconsistent across previous studies.^14^ For instance, Cai et al. observed a J-shaped association in a regional Chinese population. Specifically, compared with participants with a TyG index <9.83, those with TyG index levels ≥9.83 had 86% and 141% greater risks of all-cause and cardiovascular death, respectively.^15^ However, several analyses based on the NHANES cohort reported U-shaped relationships in which both low and high TyG index levels were associated with increased mortality.^16^^,^^18^ In addition, based on two large prospective Swedish cohorts, Muhammad et al. reported a gradual increase in participants' death risk with increasing TyG index quantiles (quantile 2: HR 1.08; quantile 3: HR 1.12; quantile 4: HR 1.22).^36^ In contrast, two Korean cohort studies revealed no significant association between the TyG index and mortality.^19^^,^^25^ Notably, in the PURE study, a global study covering 22 countries, it was reported that the TyG index was associated with cardiovascular mortality in low-income countries but not in middle- or high-income countries.^20^ Therefore, it is possible that the associations might differ across various countries and populations, and large-scale epidemiological and clinical evidence from general populations in the Western Pacific is still limited.

The reasons for the inconsistent results among the studies might be multiple and could be explained as follows. First, potential racial disparities might exist. Jevtovic et al. revealed racial differences in mitochondrial metabolism and IR; compared with Caucasians, African Americans are susceptible to reduced mitochondrial oxidative capacity and metabolic inflexibility, which are crucial risk factors for IR. Because of the vulnerability to IR, the risk and health burden of cardiometabolic diseases are greater in African Americans than in Caucasians.^37^ Potential racial metabolic differences might also exist between Chinese and American individuals, which might introduce inconsistent results across previous studies. Second, disparities in economic status across LMICs and high-income countries might also contribute to the inconsistent results. People living in LMICs are more likely to be exposed to famine and low birth weight at an early age. However, during adulthood, they might experience substantial economic development, urbanization, and modern lifestyle changes. The mismatch of starvation conditions at an early age and unhealthy lifestyles in adulthood might contribute to the vulnerability to obesity and cardiometabolic disorders such as IR.^20^^,^^38^ Therefore, it is rational that the associations between the TyG index and mortality differed across countries with various economic development statuses. Third, given that the TyG index is only associated with substantial excess mortality when above the cut-off value, the negative results in previous studies might be partially attributed to the relatively limited sample sizes of participants with a TyG index over the cut-off values, rendering the sample size insufficient for the detection of subtle risk differences.

In the current analyses, based on a national prospective cohort of more than 4 million participants, we provided novel evidence of the prognostic value of the TyG index for all-cause and cardiovascular mortality in the general population. We observed that the associations of the TyG index with all-cause and cardiovascular mortality were reverse L-shaped. Specifically, a higher TyG index indicates substantially increasing outcome risks only if the TyG index is above the cut-off value. Conversely, an elevated TyG index was not shown to be significantly associated with all-cause mortality and was related to only slightly greater cardiovascular mortality when the TyG index was below the cut-off value. In addition, the associations were similar in most subgroup analyses. The reverse L-shaped associations indicate that greater IR severity could substantially increase all-cause and cardiovascular mortality when over the thresholds.

According to our subgroup analyses, a decreasing TyG index was associated with increased risks of all-cause and cardiovascular death among females and participants with LDL-C < 55 mg/dL. This difference might be attributed to nutritional risks. Given that the TyG index was positively associated with fasting TG and glucose levels, it is possible that individuals with a low TyG index were vulnerable to undernutrition. Moreover, in China, it has been recently reported that females have lower body mass index (BMI) and visceral adiposity index (VAI) values than males do.^39^^,^^40^ Moreover, individuals with low LDL-C levels were also shown to be prone to underlying malnutrition.^41^ Therefore, among people at high risk of underweight or malnutrition, such as females and individuals with low LDL-C levels, potential nutritional deficiencies might be more concerning than metabolic risks related to IR, and a modestly elevated TyG index (lower than the cut-off value) might indicate better survival.

Compared with the outcomes of all-cause and cardiovascular mortality, evidence of the associations between the TyG index and cancer mortality among the general population is relatively scarce. Only two studies reported nonsignificant adverse associations between the TyG index and cancer mortality.^16^^,^^42^ Leveraging our large sample size, we first revealed that a 1-unit increase in the TyG index was associated with an approximately 3% lower risk of cancer death. The mechanism underlying this association remains unclear. It is possible that the TyG index is associated with a high obesity risk,^6^ and obesity is further paradoxically associated with cancer survival. Specifically, obese patients with cancer have better nutritional status and could further have better survival than their counterparts.^43^ Further investigations are still necessary to thoroughly understand the prognostic implications of the TyG index for cancer. Nevertheless, considering that excess all-cause mortality is associated with elevated TyG index levels, the TyG index cannot be regarded as a protective factor among the general population.

The underlying mechanisms and detailed roles of IR in the initiation and progression of CVDs are multiple and complicated, and several potential mechanisms have been proposed. First, IR can increase reactive oxygen species levels and reduce the levels of endothelial nitric oxide synthetase, which ultimately induces oxidative stress and exacerbates cardiometabolic disorders.^44^ Second, IR is a pathophysiological condition complicated by systemic metabolic dysfunction, which potentially triggers proinflammatory and hypertensive conditions and perturbs insulin signalling.^2^^,^^45^ These two mechanisms could further 1) recruit and activate macrophages; 2) accelerate vascular smooth muscle cell proliferation and migration; and 3) promote the apoptosis of macrophages, endothelial cells, and vascular smooth muscle cells, which all accelerate atherosclerotic plaque progression.^2^ Third, IR can also enhance collagen deposition and promote cardiac and vascular stiffness, which ultimately results in heart failure and peripheral vascular diseases.^46^^,^^47^ Fourth, IR can also lead to platelet dysfunction, including activation, hyperaggregation, and adhesion to endothelial cells, which further enhances vasoconstriction and promotes thrombus formation.^48^

### Implication

Along with the awareness, treatment, and control of traditional cardiovascular risk factors, such as LDL-C concentrations, residual risk factors and further risk stratification have become increasingly important.^49^^,^^50^ We found that the association between the TyG index and mortality is independent of conventional CVD risk factors, such as LDL-C and diabetes. With these findings, the TyG index could serve as an effective tool for identifying people at high risk of all-cause and cardiovascular death, and all people with TyG index levels above the cut-off value of 9.75 should be given targeted attention and health interventions, regardless of their current ASCVD risk stratification, age, central obesity status, LDL-C levels, or medical history of hypertension and diabetes. In addition, given that the TyG index can be easily calculated using fasting glucose and TG levels, the TyG index could be a simple and convenient screening tool applicable in routine clinical practice, even in resource-limited settings, especially LMICs. Therefore, to achieve precise risk stratification for CVD patients, identify individuals with IR who require medical intervention, and improve the survival and cardiovascular health of those individuals in a targeted manner, a policy promoting TyG index assessment in a large population may be a beneficial and cost-effective approach.

### Strengths and limitations

Our study has several strengths. First, this is the largest study to investigate the associations between the TyG index and mortality. With the large sample size of 3.5 million and the geographic and demographic diversity of the participants, we first provided evidence of the prognostic implications of the TyG index on all-cause, cardiovascular, and cancer mortality in the Chinese general population, which could also extend the clinical evidence in Asian and LMIC peoples. The large sample size also provided an opportunity to thoroughly estimate these associations in detailed subgroup analyses. However, our findings should be interpreted in the context of several limitations. First, in the present study, approximately 20% of participants were excluded due to a lack of data, and the excluded individuals were more likely to be males, which might introduce selection bias into the results. Nevertheless, we performed subgroup analyses by sex, and the results were similar. Therefore, our results were robust. Second, given the observational nature, residual confounding bias due to unmeasured factors, such as diet and physical activity, could not be completely ruled out. For example, foods rich in dietary fibre (e.g., legumes, vegetables, and whole grains) are beneficial for reducing the risk of IR, which could further protect cardiovascular health. Similarly, regular exercise, including an active lifestyle and structured physical activity, could also improve IR status. The lack of data might have led to underestimation of the association between the TyG index and cardiovascular outcomes among participants with diets rich in fibre or active exercise.^51^ Third, we collected data on only the TyG index and covariates at baseline, and we could not account for the longitudinal changes during the follow-up. Fourth, the follow-up duration was relatively short (median <5 years), which might have led to insufficient power to examine the long–term association between the TyG index and mortality. However, in this circumstance, the TyG index at baseline could be a better marker to reflect the IR of participants during follow-up, which could help to evaluate the real association with outcomes even more closely. Finally, all participants were Chinese, and caution is needed when generalizing the conclusions to other races or countries.

### Conclusions

In a nationwide cohort study in China, we observed reverse L-shaped associations between the TyG index and all-cause and cardiovascular mortality. Specifically, an elevated TyG index above 9.75 and 9.85, but not below these cut-off values, was associated with markedly increased all-cause and cardiovascular mortality, respectively. These findings suggested that the TyG index could serve as a simple tool to assist physicians in screening people vulnerable to all-cause and cardiovascular death, emphasizing the necessity for targeted attention and treatment for people with a TyG index exceeding 9.75.

## Contributors

GH, ZZ, CW, and LC contributed to the conceptualisation of the study. CW, XB, and XZ performed data curation. GH, ZZ, and CW contributed to formal analysis. LC contributed to funding acquisition. XZ, JC, WX, LS, HY, WH, and YZ contributed to investigation. GH, ZZ, CW, XB, LH, SC, GL, and LC contributed to methodology. YY and XL contributed to project administration. XL and LC contributed to resources. GH, XB, and LH contributed to software. XL and LC contributed to supervision. XB and LH contributed to validation. GH contributed to visualisation. GH originally drafted the manuscript, and all authors (GH, ZZ, CW, WW, XB, LH, SC, GL, YY, XZ, JC, WX, LS, HY, WH, YZ, XL, and LC) contributed to writing-review & editing. All authors have read and approved the final manuscript. The corresponding author attests that all listed authors meet authorship criteria and that no others meeting the criteria have been omitted.

## Data sharing statement

The data are not publicly available. ChinaHEART only provides conditional data access for qualified researchers with legitimate requests; a formal application and research proposal are required. Please contact cvd-project@nccd.org.cn to seek approval for data access.

## Declaration of interests

None.
