# Supplementary material for: Association of the triglyceride–glucose index with all-cause and cause-specific mortality: a population-based cohort study of 3.5 million adults in China
Source: Lancet Reg Health West Pac. 2024 Jul 6;49:101135. doi: 10.1016/j.lanwpc.2024.101135 (PMC11263946; doi:10.1016/j.lanwpc.2024.101135)
Supplement: Supplement Figures [file mmc1.docx]

**SUPPLEMENTAL MATERIAL**

**Part 1. Systematic review and novelty.**

**Table S1. Participant characteristics among included and excluded participants.**

**Figure S1. Adjusted association between triglyceride-glucose index and mortality by arteriosclerotic cardiovascular disease risk stratification.**

**Figure S2. Adjusted association between triglyceride-glucose index and mortality by sex.**

**Figure S3. Adjusted association between triglyceride-glucose index and mortality by age quartiles.**

**Figure S4. Adjusted association between triglyceride-glucose index and mortality by central obesity.**

**Figure S5. Adjusted association between triglyceride-glucose index and mortality by low-density lipoprotein cholesterol subgroups.**

**Figure S6. Adjusted association between triglyceride-glucose index and mortality by hypertension.**

**Figure S7. Adjusted association between triglyceride-glucose index and mortality by diabetes.**

**Figure S8. Adjusted association between triglyceride-glucose index and mortality by antidiabetic drugs among participants with diabetes.**

**Figure S9. Sensitivity analyses of adjusting for non-high-density lipoprotein cholesterol.**

**Figure S10. Sensitivity analyses among participants without using lipid-lowering drugs.**

**Part 1. Systematic review and novelty.**

The triglyceride-glucose (TyG) index, developed in 2008, serves as a sensitive surrogate biomarker of insulin resistance (IR).^1^ Unlike direct measurements of IR, such as the homeostasis model assessment of insulin resistance (HOMA-IR), the TyG index offers simplicity and cost-effectiveness in daily clinical practice. Numerous studies have demonstrated that an elevated TyG index is a risk factor for cardiovascular and metabolic diseases.^2-4^ However, most previous studies were based on patients with specific cardiovascular or metabolic diseases (e.g., coronary heart disease, type 2 diabetes, and heart failure) or focused on the incidence of these diseases.^4^ The evidence linking the TyG index to mortality in the general population remains relatively limited.

We searched PubMed for “TyG index” or “triglyceride glucose index” during May 2024. After excluding studies focusing on patients with specific cardiovascular or metabolic diseases, we included all studies based on general populations that assessed the associations between the TyG index and all-cause and cause-specific mortality. Ultimately, 20 population-based cohort studies were included and are summarized in the table in **Supplement Part 1**. Most of these studies indicated that an elevated TyG index was associated with increased risks of all-cause and cardiovascular mortality. Participants in higher tertiles/quartiles of the TyG index exhibited greater vulnerability to mortality. However, the results of these studies still exhibit discrepancies. Among 8 studies that evaluated nonlinear associations, six studies conducted in US participants (using data from the National Health and Nutrition Examination Survey [NHANES] database) reported U-, J-, or reverse L-shaped associations,^5-10^ one Korean study identified a U-shaped association with all-cause mortality but a J-shaped association with cardiovascular mortality,^11^ while one Chinese study observed a J-shaped association.^12^ Several potential factors could contribute to the inconsistent results, including racial differences, variations in metabolism, social and economic development status, and environmental exposure.

First, there are racial and metabolic differences among various populations, and those in the Western Pacific region were underrepresented in previous studies. Jevtovic et al. revealed that compared with Caucasians, African Americans are susceptible to lower mitochondrial oxidative capacity and metabolic inflexibility, and such differences in metabolism among races potentially contribute to their vulnerability to IR.^13^ Given these variations across populations, findings from studies conducted primarily on Caucasians may not accurately reflect the relationship between the TyG index and mortality in other ethnic groups. Moreover, most previous evidence was based on US population databases, particularly the NHANES database, and only a few studies were conducted on other people. Consequently, the generalizability of these findings to populations in the Western Pacific region, such as the Chinese region, remains uncertain. Additionally, the only large-scale population-based study conducted in China included only participants at high cardiovascular risk,^12^ who are more susceptible to metabolic risk factors such as diabetes, dyslipidaemia, and obesity.^12,14^ Therefore, the existing results may not fully capture the prognostic implications of the TyG index for mortality in the general population.

Second, socioeconomic status may influence the association between the TyG index and mortality. An analysis of the Prospective Urban Rural Epidemiology (PURE) study, which involved participants from 22 countries, reported that the TyG index only significantly affected cardiovascular mortality in low-income and middle-income countries (LMICs) but not in high-income countries.^15^ One possible explanation is that individuals in LMICs may experience maternal undernutrition at an early age, but as economic development and urbanization progress, they subsequently experience modern lifestyle changes (e.g., low physical activity and an obesogenic diet) in adulthood. The mismatch of starvation conditions at an early age and unhealthy lifestyles in adulthood might contribute to the vulnerability of individuals living in LMICs to IR.^15^ However, most of the prior studies were conducted in developed and high-income countries, such as the US, Korea, and Sweden, which could lead to the underrepresentation of LMICs. Therefore, conducting studies in LMICs is essential for a comprehensive understanding of the implications of the TyG index for mortality.

Third, recent studies have suggested that exposure to environmental factors, such as fine particulate matter (PM_2.5_) and ozone (O_3_), could increase the risk of IR.^16,17^ China, with its vast territory in the Western Pacific region, exhibits diverse environmental characteristics.^18,19^ However, the only large-scale study of the TyG index and mortality in the Chinese population has been limited to a single province.^12^ Given the environmental diversity across China, a nationwide study covering the entire country may be necessary to assess the implications of the TyG index for the general Chinese population.

In summary, previous studies examining the association between the TyG index and mortality may not have adequately represented the populations in the Western Pacific region. To comprehensively understand the prognostic implications of the TyG index for mortality in the general population, we conducted analyses based on a national prospective cohort study. With the large sample size and the geographic and demographic diversity of the participants, we found reverse L-shaped associations of the TyG index with all-cause and cardiovascular mortality and a slightly negative association between the TyG index and cancer mortality. The associations were similar across various subgroups. The reverse L-shaped associations indicate that individuals with elevated TyG index levels warrant targeted medical intervention. Our study provided novel evidence of the prognostic value of the TyG index for survival in the general population in Asia and LMICs. The importance of residual cardiovascular risk assessment has emerged in recent years. Given the simplicity of measurement, in addition to conventional cardiovascular and metabolic risk factors, such as low-density lipoprotein cholesterol (LDL-C) concentrations, the TyG index could serve as a cost-effective tool for screening individuals vulnerable to all-cause and cardiovascular death in large-scale populations.

**Supplement part 1 table. Previous studies focused on the association of triglyceride-glucose index with mortality among population-based participants.**

| **Year** | **Author** | **Database (Country)** | **Sample size** | **TyG index** | **Follow-up** | **Association with mortality** |
| --- | --- | --- | --- | --- | --- | --- |
| 2024 | Wei X,  et al^20^ | NHANES (US) | 10734 | Quartiles | median  100 months | ACM: Q4 (Ref Q1) HR: 1.36 (1.18, 1.56)  CVM: Q4 (Ref Q1) HR: 1.29 (0.99, 1.69)  Diabetes mortality: Q4 (Ref Q1) HR: 4.06 (2.81, 5.87) |
| 2024 | Du L, et al^21^ | NHANES (US) | 6731 | Quartiles | 16.7 years | ACM (Ref Q3):  Q1 HR: 1.32 (1.03, 1.70); Q2 HR: 1.25 (1.01, 1.55) CVM (Ref Q3):  Q1 HR: 0.73 (0.39, 1.36); Q2 HR: 0.55 (0.32, 0.93) |
| 2024 | Alavi Tabatabaei G, et al^22^ | Iranian population-based study (Iran) | 5432 | Tertiles | median  11.25 years | ACM: T3 (Ref T1) HR: 1.41 (1.11, 1.81)  CVM: T3 (Ref T1) HR: 1.64 (1.07, 2.50) Both the associations were negative when further adjusted for diabetes. |
| 2023 | Cai X, et al^12^ | Population-based participants with high CV risk (China) | 35455 | TyG≥9.83 | median  3.4 years | J-shaped associations with ACM and CVM  Cut-off value: ACM=9.83; CVM=10.05  ACM: TyG≥9.83 (Ref TyG <9.83) HR: 1.86 (1.37, 2.51) CVM: TyG≥9.83 (Ref TyG <9.83) HR: 2.41 (1.47, 3.96) |
| 2022 | Xu X, et al^23^ | CARDIA (US) | 4754 | Per 1-unit | median  25 years | ACD: HR: 1.96 (1.44, 2.66)  CVD: HR: 1.85 (1.45, 2.36) |
| 2023 | Yu Y, et al^5^ | NHANES (US) | 7851 | Per 1-unit | 11623  person-years | U-shaped associations with ACM and CVM Cut-off value: ACM=8.5; CVM=8.7  ACM: TyG<8.5: HR: 0.55 (0.34, 0.90); TyG≥8.5: HR: 1.39 (1.14, 1.70)  CVM: TyG<8.7: HR: 0.30 (0.14, 0.65); TyG≥8.7: HR: 1.82 (1.14, 2.92) |
| 2023 | Li H, et al^6^ | NHANES (US) | 2072 | Quartiles | 16.8 years | U-shaped association with ACM  Lowest risk: 8.83≤TyG≤9.06  ACM:  Q1 (Ref quartile [Q3]): HR: 1.40 (1.13, 1.74)  Q4 (Ref quartile [Q3]): HR: 1.08 (0.88, 1.32)  No significant association of TyG index and CVM. |
| 2023 | Chen W,  et al^7^ | NHANES (US) | 16613 | TyG≥8.5 | median  123 months | J-shaped association with ACM  Cut-off value: ACM=8.5  ACM: HR: 1.32 (1.07,1.63) |
| 2023 | Anthony Kityo, et al^11^ | Korean Genome and Epidemiology Study (Korea) | 114957 | Continuous variable | 1217002  person-years | U-shaped association with ACM  J-shaped association with CVM  No significant association with cancer mortality |
| 2023 | Chen J, et al^8^ | NHANES (US) | 20194 | Continuous variable | median  105 months | Reverse L-shaped associations with ACM and CVM  ACM: HR: 1.160 (1.062, 1.267)  CVM: HR: 1.213 (1.020, 1.441) |
| 2023 | Lopez-Jaramillo P, et al^15^ | PURE Study  (22 countries) | 141243 | Tertiles | median  13.2 years | Significant association only in low-income and middle-income countries CVM: T3 (Ref T1) HR: 1.44 (1.15-1.80) |
| 2022 | Tian X,  et al^24^ | Kailuan study (China) | 51734 | TyG slope &  CumTyG | median  9.04 years | TyG slope<0 (Ref TyG slope≥0):  ACM: HR: 1.18 (1.10–1.26) CVM: HR: 1.11 (1.04–1.19) CumTyG≥median and TyG slope<0 (Ref cumTyG<median and TyG slope≥0):  ACM: HR: 1.28 (1.15, 1.43) CVM: HR: 1.37 (1.24, 1.51) |
| 2022 | Yu Y, et al^25^ | NHANES (US) | 6245 | For ACM: TyG<8.86  For CVM: TyG<8.55 | median  66.8 months | ACM (Ref TyG≥8.86): HR: 0.55 (0.39, 0.78)  CVM (Ref TyG≥8.86): HR: 0.58 (0.31, 0.96) |
| 2022 | Mirshafiei H, et al^26^ | MASHAD study (Iran) | 9704 | Per 1-unit | 6 years | Sudden death: OR: 2.339 (1.247, 4.241) |
| 2022 | Kim KS,  et al^27^ | Kangbuk Samsung Health Study (Korea) | 255508 | Quartiles | median  5.7 years | ACM:  Overall Q4 (Ref: Q1) HR: 0.97 (0.90, 1.03) Men Q4 (Ref: Q1): HR: 0.92 (0.85, 0.99)  Women Q4 (Ref: Q1): HR: 1.13 (1.02, 1.26)  CVM:  Overall Q4 (Ref: Q1): HR: 1.04 (0.88, 1.25) Men Q4 (Ref: Q1): HR: 1.03 (0.84, 1.26)  Women Q4 (Ref: Q1): HR: 1.04 (0.77, 1.39) |
| 2022 | Cho YK,  et al^28^ | NHIS-HEALS (Korea) | 292206 | Quartiles | <6 years | Metabolically unhealthy obese:  Q4 (Ref Q1) HR: 1.48 (1.13, 1.93) |
| 2022 | Muhammad IF, et al^29^ | Malmö Preventive project (Sweden) | 32960 | Quartiles | median  21.2 years | ACM (Ref: Q1):  Q2 HR: 1.08 (1.04–1.13); Q3 HR: 1.12 (1.07–1.17); Q4 HR: 1.22 (1.16–1.28) CVM (Ref: Q1):  Q2 HR: 1.11 (1.02–1.20); Q3 HR: 1.19 (1.10–1.29); Q4 HR: 1.37 (1.26–1.49) |
| 2022 | Sun M, et al^9^ | NHANES (US) | 9254 | Per 1-unit  &  Quartiles | median  7.6 years | U-shaped associations with ACM  Cut-off for ACM: 9.18 ACM: <9.18 HR (per 1-unit): 0.82 (0.71, 0.96);  ACM: >9.18 HR (per 1-unit): 1.32 (1.12,1.55)  ACM: Q3 (Ref: Q1) HR=0.84 (0.73, 0.98) CVM: Q2 (Ref: Q1) HR=0.62 (0.43, 0.88) Malignant neoplasms mortality: Not significant |
| 2021 | Kim J, et al^30^ | NHIS-HEALS (Korea) | 114603 | Quartiles | median  5.97 years | No significant association with ACM or CVM |
| 2020 | Liu XC,  et al^10^ | NHANES (US) | 19420 | Per 1-unit | mean  98.2 months | J-shaped associations with ACM and CVM  Cut-off value: ACM=9.36; CVM=9.52  ACM HR: 1.10 (1.00, 1.20)  CVM: HR: 1.29 (1.05, 1.57) |

Abbreviation: TyG index: triglyceride-glucose index; NHANES: National Health and Nutrition Examination Survey; CV: cardiovascular; ACM: all-cause mortality; CVM: cardiovascular mortality; Q1: quartile 1; Q4: quartile 4; Q2: quartile 2; Q3: quartile 3; T1: tertile 1; T3: tertile 3; HR: hazard ratio; CI: confidence interval; CARDIA: Coronary Artery Risk Development in Young Adults study; PURE: Prospective Urban Rural Epidemiology; CumTyG: cumulative triglyceride-glucose index; OR: odds ratio; NHIS-HEALS: Korean National Health Insurance Service-National Health Screening Cohort.

Reference:

1. Simental-Mendía LE, Rodríguez-Morán M, Guerrero-Romero F. The product of fasting glucose and triglycerides as surrogate for identifying insulin resistance in apparently healthy subjects. *Metab Syndr Relat Disord* 2008;**6**:299-304. doi: 10.1089/met.2008.0034

2. Liang S, Wang C, Zhang J*, et al.* Triglyceride-glucose index and coronary artery disease: a systematic review and meta-analysis of risk, severity, and prognosis. *Cardiovasc Diabetol* 2023;**22**:170. doi: 10.1186/s12933-023-01906-4

3. Khalaji A, Behnoush AH, Khanmohammadi S*, et al.* Triglyceride-glucose index and heart failure: a systematic review and meta-analysis. *Cardiovasc Diabetol* 2023;**22**:244. doi: 10.1186/s12933-023-01973-7

4. Tao LC, Xu JN, Wang TT, Hua F, Li JJ. Triglyceride-glucose index as a marker in cardiovascular diseases: landscape and limitations. *Cardiovasc Diabetol* 2022;**21**:68. doi: 10.1186/s12933-022-01511-x

5. Yu Y, Wang J, Ding L*, et al.* Sex differences in the nonlinear association of triglyceride glucose index with all-cause and cardiovascular mortality in the general population. *Diabetol Metab Syndr* 2023;**15**:136. doi: 10.1186/s13098-023-01117-7

6. Li H, Jiang Y, Su X, Meng Z. The triglyceride glucose index was U-shape associated with all-cause mortality in population with cardiovascular diseases. *Diabetol Metab Syndr* 2023;**15**:181. doi: 10.1186/s13098-023-01153-3

7. Chen W, Ding S, Tu J*, et al.* Association between the insulin resistance marker TyG index and subsequent adverse long-term cardiovascular events in young and middle-aged US adults based on obesity status. *Lipids Health Dis* 2023;**22**:65. doi: 10.1186/s12944-023-01834-y

8. Chen J, Wu K, Lin Y, Huang M, Xie S. Association of triglyceride glucose index with all-cause and cardiovascular mortality in the general population. *Cardiovasc Diabetol* 2023;**22**:320. doi: 10.1186/s12933-023-02054-5

9. Sun M, Guo H, Wang Y, Ma D. Association of triglyceride glucose index with all-cause and cause-specific mortality among middle age and elderly US population. *BMC Geriatr* 2022;**22**:461. doi: 10.1186/s12877-022-03155-8

10. Liu XC, He GD, Lo K, Huang YQ, Feng YQ. The Triglyceride-Glucose Index, an Insulin Resistance Marker, Was Non-linear Associated With All-Cause and Cardiovascular Mortality in the General Population. *Front Cardiovasc Med* 2020;**7**:628109. doi: 10.3389/fcvm.2020.628109

11. Kityo A, Lee SA. Association of cardiometabolic factors and insulin resistance surrogates with mortality in participants from the Korean Genome and Epidemiology Study. *Lipids Health Dis* 2023;**22**:210. doi: 10.1186/s12944-023-01981-2

12. Cai XL, Xiang YF, Chen XF*, et al.* Prognostic value of triglyceride glucose index in population at high cardiovascular disease risk. *Cardiovasc Diabetol* 2023;**22**:198. doi: 10.1186/s12933-023-01924-2

13. Jevtovic F, Krassovskaia PM, Lopez CA*, et al.* Mitochondrial Phenotype as a Driver of the Racial Dichotomy in Obesity and Insulin Resistance. *Biomedicines* 2022;**10**. doi: 10.3390/biomedicines10061456

14. Lu J, Lu Y, Yang H*, et al.* Characteristics of High Cardiovascular Risk in 1.7 Million Chinese Adults. *Ann Intern Med* 2019;**170**:298-308. doi: 10.7326/m18-1932

15. Lopez-Jaramillo P, Gomez-Arbelaez D, Martinez-Bello D*, et al.* Association of the triglyceride glucose index as a measure of insulin resistance with mortality and cardiovascular disease in populations from five continents (PURE study): a prospective cohort study. *Lancet Healthy Longev* 2023;**4**:e23-e33. doi: 10.1016/s2666-7568(22)00247-1

16. Zhang Z, Luan C, Wang C*, et al.* Insulin resistance and its relationship with long-term exposure to ozone: Data based on a national population cohort. *J Hazard Mater* 2024;**472**:134504. doi: 10.1016/j.jhazmat.2024.134504

17. Zhao L, Fang J, Tang S*, et al.* PM2.5 and Serum Metabolome and Insulin Resistance, Potential Mediation by the Gut Microbiome: A Population-Based Panel Study of Older Adults in China. *Environ Health Perspect* 2022;**130**:27007. doi: 10.1289/ehp9688

18. Meng X, Wang W, Shi S*, et al.* Evaluating the spatiotemporal ozone characteristics with high-resolution predictions in mainland China, 2013-2019. *Environ Pollut* 2022;**299**:118865. doi: 10.1016/j.envpol.2022.118865

19. Geng G, Xiao Q, Liu S*, et al.* Tracking Air Pollution in China: Near Real-Time PM(2.5) Retrievals from Multisource Data Fusion. *Environ Sci Technol* 2021;**55**:12106-12115. doi: 10.1021/acs.est.1c01863

20. Wei X, Min Y, Song G, Ye X, Liu L. Association between triglyceride-glucose related indices with the all-cause and cause-specific mortality among the population with metabolic syndrome. *Cardiovasc Diabetol* 2024;**23**:134. doi: 10.1186/s12933-024-02215-0

21. Du L, Xu X, Wu Y, Yao H. Association between the triglyceride glucose index and cardiovascular mortality in obese population. *Nutr Metab Cardiovasc Dis* 2024;**34**:107-111. doi: 10.1016/j.numecd.2023.08.007

22. Alavi Tabatabaei G, Mohammadifard N, Rafiee H*, et al.* Association of the triglyceride glucose index with all-cause and cardiovascular mortality in a general population of Iranian adults. *Cardiovasc Diabetol* 2024;**23**:66. doi: 10.1186/s12933-024-02148-8

23. Xu X, Huang R, Lin Y*, et al.* High triglyceride-glucose index in young adulthood is associated with incident cardiovascular disease and mortality in later life: insight from the CARDIA study. *Cardiovasc Diabetol* 2022;**21**:155. doi: 10.1186/s12933-022-01593-7

24. Tian X, Chen S, Zhang Y*, et al.* Time course of the triglyceride glucose index accumulation with the risk of cardiovascular disease and all-cause mortality. *Cardiovasc Diabetol* 2022;**21**:183. doi: 10.1186/s12933-022-01617-2

25. Yu Y, Gu M, Huang H*, et al.* Combined association of triglyceride-glucose index and systolic blood pressure with all-cause and cardiovascular mortality among the general population. *J Transl Med* 2022;**20**:478. doi: 10.1186/s12967-022-03678-z

26. Mirshafiei H, Darroudi S, Ghayour-Mobarhan M*, et al.* Altered triglyceride glucose index and fasted serum triglyceride high-density lipoprotein cholesterol ratio predict incidence of cardiovascular disease in the Mashhad cohort study. *Biofactors* 2022;**48**:643-650. doi: 10.1002/biof.1816

27. Kim KS, Hong S, Hwang YC, Ahn HY, Park CY. Evaluating Triglyceride and Glucose Index as a Simple and Easy-to-Calculate Marker for All-Cause and Cardiovascular Mortality. *J Gen Intern Med* 2022;**37**:4153-4159. doi: 10.1007/s11606-022-07681-4

28. Cho YK, Kim HS, Park JY*, et al.* Triglyceride-Glucose Index Predicts Cardiovascular Outcome in Metabolically Unhealthy Obese Population: A Nationwide Population-Based Cohort Study. *J Obes Metab Syndr* 2022;**31**:178-186. doi: 10.7570/jomes21086

29. Muhammad IF, Bao X, Nilsson PM, Zaigham S. Triglyceride-glucose (TyG) index is a predictor of arterial stiffness, incidence of diabetes, cardiovascular disease, and all-cause and cardiovascular mortality: A longitudinal two-cohort analysis. *Front Cardiovasc Med* 2022;**9**:1035105. doi: 10.3389/fcvm.2022.1035105

30. Kim J, Shin SJ, Kang HT. The association between triglyceride-glucose index, cardio-cerebrovascular diseases, and death in Korean adults: A retrospective study based on the NHIS-HEALS cohort. *PLoS One* 2021;**16**:e0259212. doi: 10.1371/journal.pone.0259212

**Table S1. Participant characteristics among included and excluded participants.**

| **Characteristic^a^** | **Overall**  **(n=4404586)** | **Included**  **(n=3524459)** | **Excluded**  **(n=880127)** |
| --- | --- | --- | --- |
| **Age, year** | 56 (48, 64) | 56 (49, 64) | 56 (48, 64) |
| **Female** | 2617703 (59.4) | 2136709 (60.6) | 480994 (54.7) |
| **Clinical characteristics** |  |  |  |
| SBP, mmHg | 134 (122, 148) | 134 (122, 148) | 134 (122, 148) |
| DBP, mmHg | 81 (74, 88) | 81 (74, 88) | 81 (74, 88) |
| Body mass index, kg/m^3^ | 24.4 (22.4, 26.8) | 24.5 (22.4, 26.9) | 24.1 (22.0, 26.3) |
| Waist, cm | 83 (78, 90) | 84 (78, 90) | 83 (82, 84)^b^ |
| Central obesity | /^b^ | 1349181 (38.3) | /^b^ |
| **Marital status** |  |  |  |
| Married | 4097285 (93.0) | 3276299 (93.0) | 820986 (93.3) |
| Unmarried | 263393 (6.0) | 211593 (6.0) | 51800 (5.9) |
| Unknown | 43908 (1.0) | 36567 (1.0) | 7341 (0.8) |
| **Education** |  |  |  |
| Primary school or lower | 1976002 (44.9) | 1576464 (44.7) | 399538 (45.4) |
| Middle school | 1409725 (32.0) | 1130784 (32.1) | 278941 (31.7) |
| High school | 635219 (14.4) | 504474 (14.3) | 130745 (14.9) |
| College or higher | 338495 (7.7) | 273881 (7.8) | 64614 (7.3) |
| Unknown | 45145 (1.0) | 38856 (1.1) | 6289 (0.7) |
| **Last year household income** |  |  |  |
| < 10000 RMB | 766432 (17.4) | 605346 (17.2) | 161086 (18.3) |
| 10,000 ≤ income < 50000 RMB | 2415383 (54.8) | 1932326 (54.8) | 483057 (54.9) |
| ≥ 50000 RMB | 797464 (18.1) | 659593 (18.7) | 137871 (15.7) |
| Unknown | 425307 (9.7) | 327194 (9.3) | 98113 (11.1) |
| **Medical insurance** |  |  |  |
| With medical insurance | 4310429 (97.9) | 3443302 (97.7) | 867127 (98.5) |
| Without medical insurance | 14194 (0.3) | 11851 (0.3) | 2343 (0.3) |
| Unknown | 79963 (1.8) | 69306 (2.0) | 10657 (1.2) |
| **Residence** |  |  |  |
| Urban | 1766962 (40.1) | 1404386 (39.8) | 362576 (41.2) |
| Rural | 2637624 (59.9) | 2120073 (60.2) | 517551 (58.8) |
| **Region** |  |  |  |
| North | 1783510 (40.5) | 1420949 (40.3) | 362561 (41.2) |
| South | 2621076 (59.5) | 2103510 (59.7) | 517566 (58.8) |
| **Current smoker** | 862141 (19.6) | 677469 (19.2) | 184672 (21.0) |
| **Current drinker** | 1042013 (23.7) | 814161 (23.1) | 227852 (25.9) |
| **Medical history** |  |  |  |
| Hypertension | 1789516 (40.6) | 1435019 (40.7) | 354497 (40.3) |
| Diabetes | 308481 (7.0) | 254353 (7.2) | 54128 (6.2) |
| COPD | 9898 (0.2) | 8331 (0.2) | 1567 (0.2) |
| Cancer | 16558 (0.4) | 14033 (0.4) | 2525 (0.3) |
| **Using lipid-lowering drugs** | 122881 (2.8) | 100549 (2.9) | 22332 (2.5) |
| **Using antidiabetic drugs** | 269272 (6.1) | 223213 (6.3) | 46059 (5.2) |

Notes: ^a^: Results are presented as interquartile range for continuous variables or number (percentage) for categorical variables; ^b^: 392224 (44.6%) individuals missed waist data among the excluded participants, therefore, we did not calculate the central obesity rate in the overall and excluded participants.

Abbreviations: SBP: systolic blood pressure; DBP: diastolic blood pressure; COPD: chronic obstructive pulmonary disease.

**Figure S1. Adjusted association between triglyceride-glucose index and mortality by arteriosclerotic cardiovascular disease risk stratification.**

**
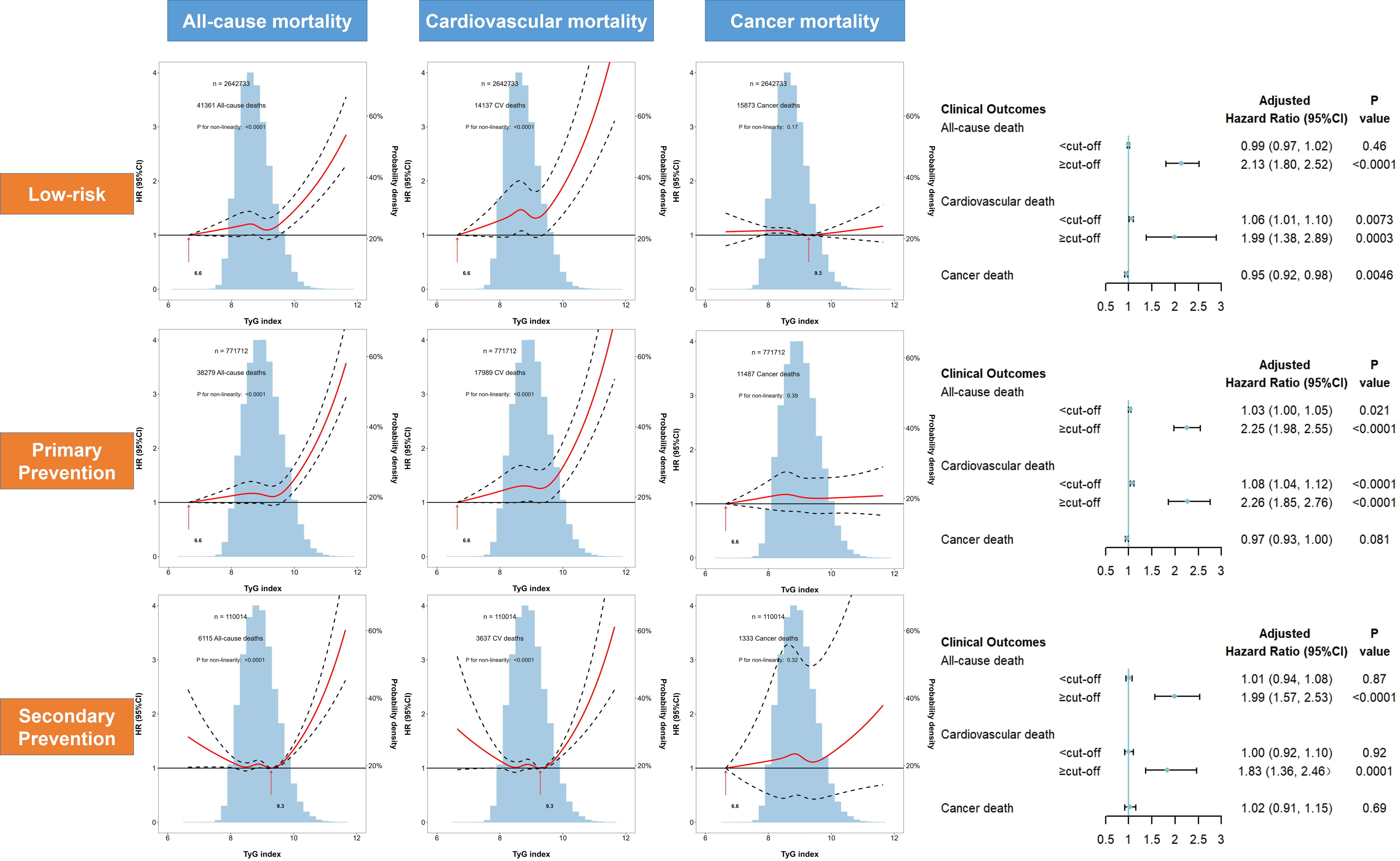
**

**Figure S2. Adjusted association between triglyceride-glucose index and mortality by sex.**

**
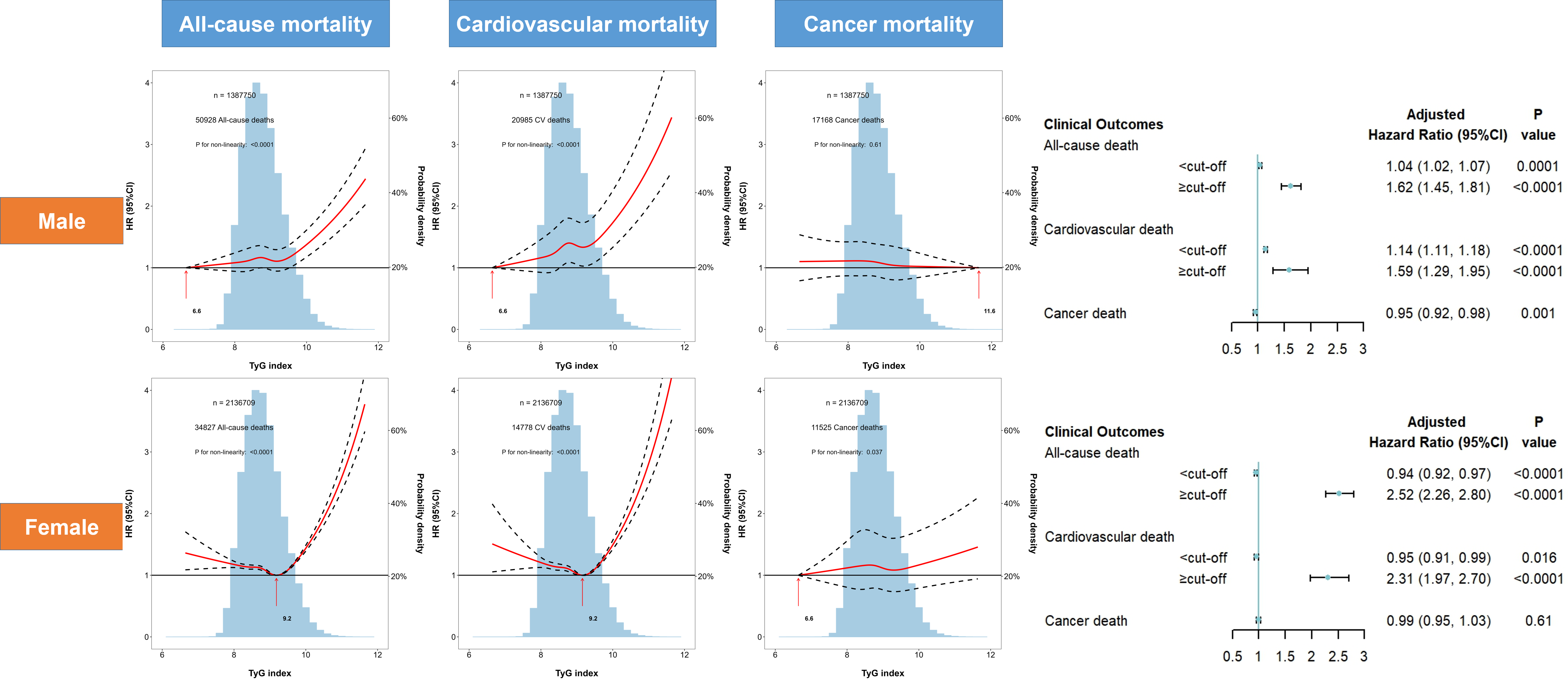
**

**Figure S3. Adjusted association between triglyceride-glucose index and mortality by age quartiles.**

**
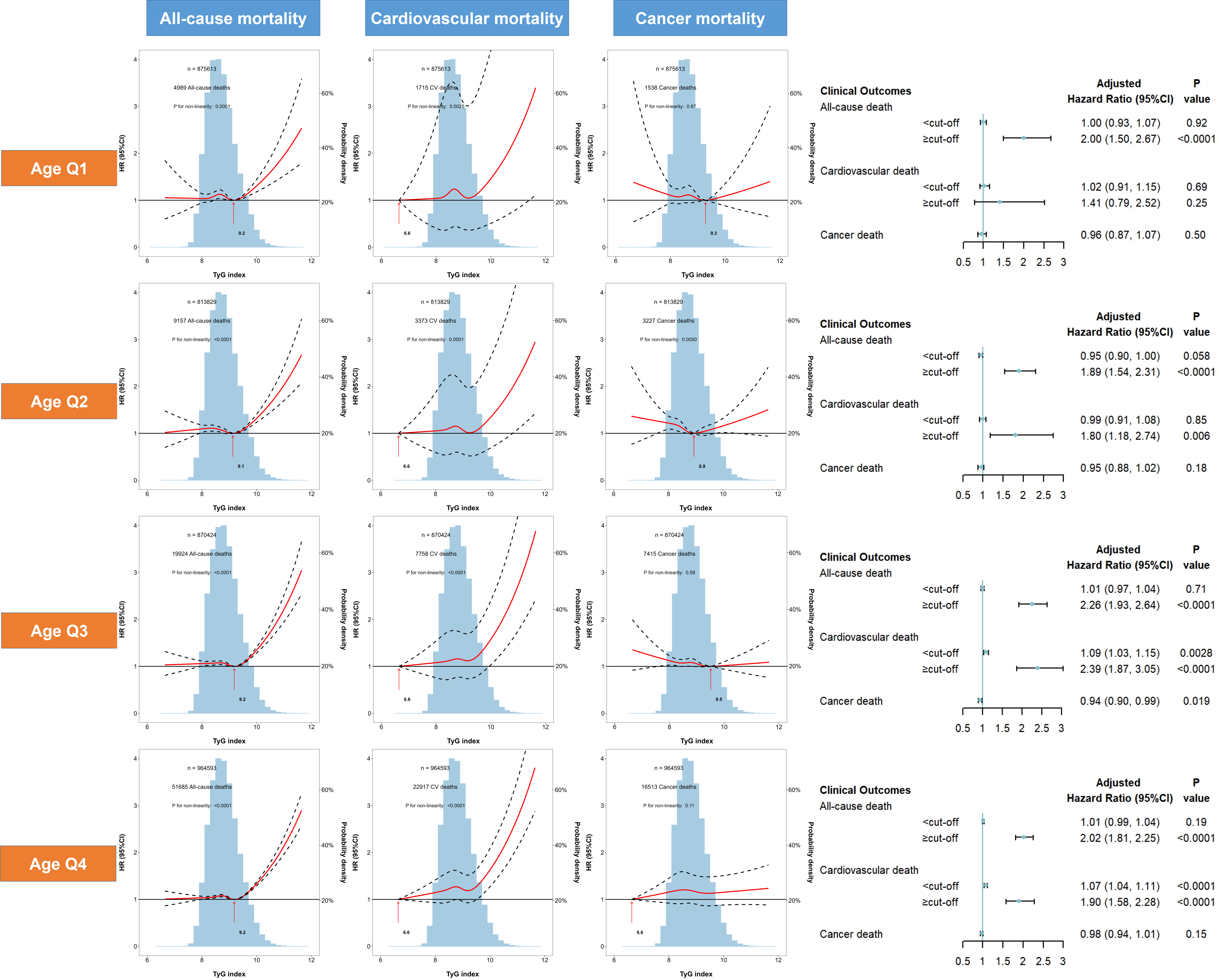
**

**Figure S4. Adjusted association between triglyceride-glucose index and mortality by central obesity.**

**
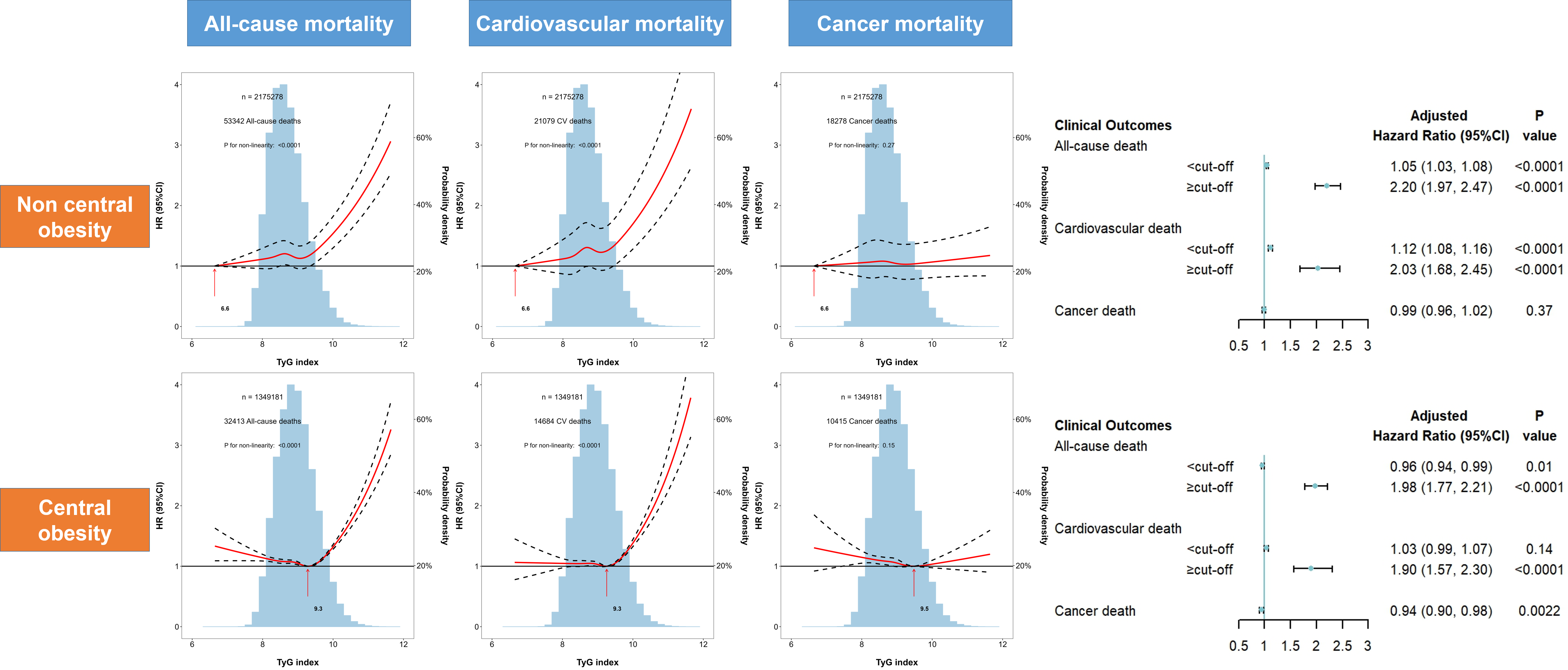
**

**Figure S5. Adjusted association between triglyceride-glucose index and mortality by low-density lipoprotein cholesterol subgroups.**

**
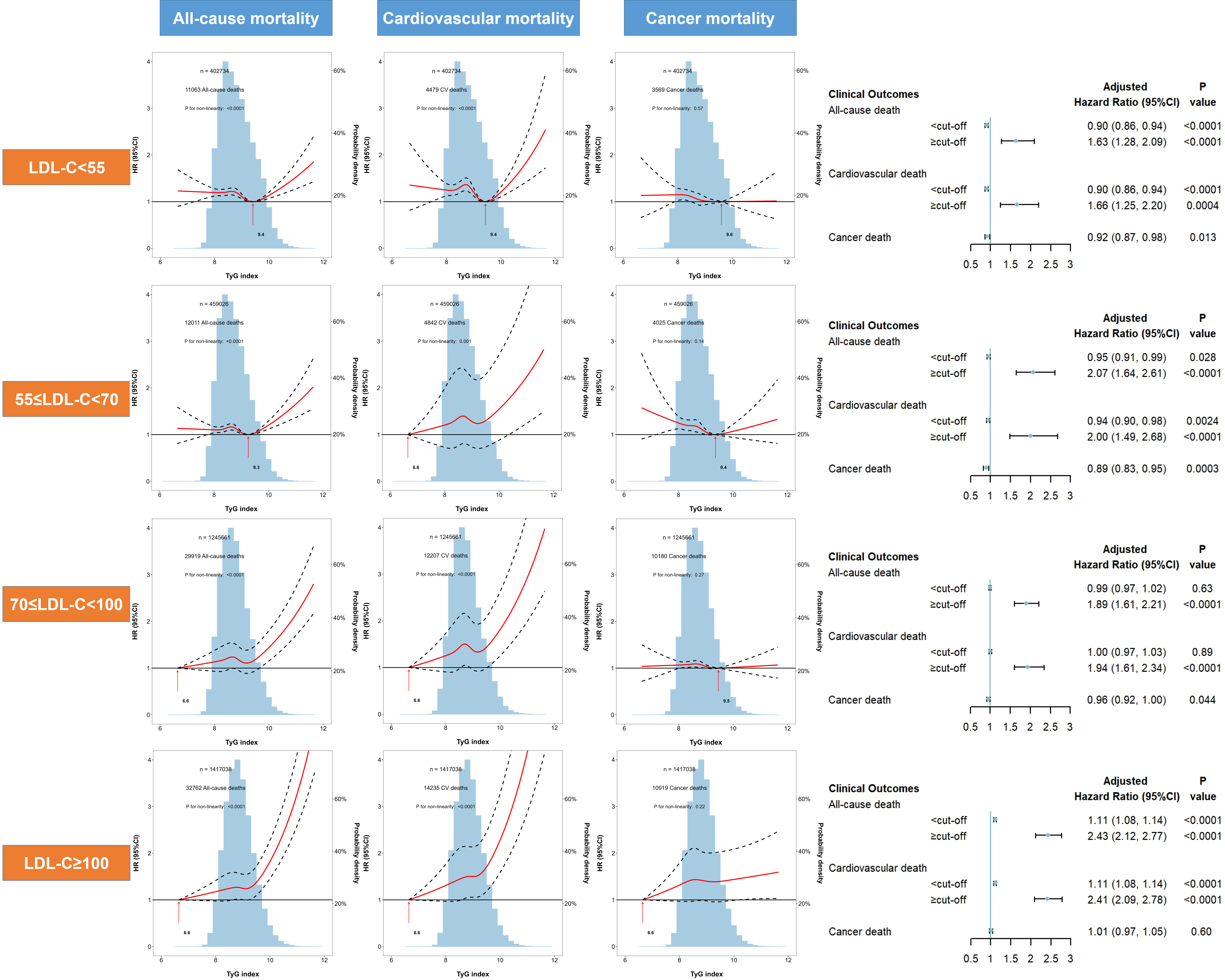
**

**Figure S6. Adjusted association between triglyceride-glucose index and mortality by hypertension.**

**
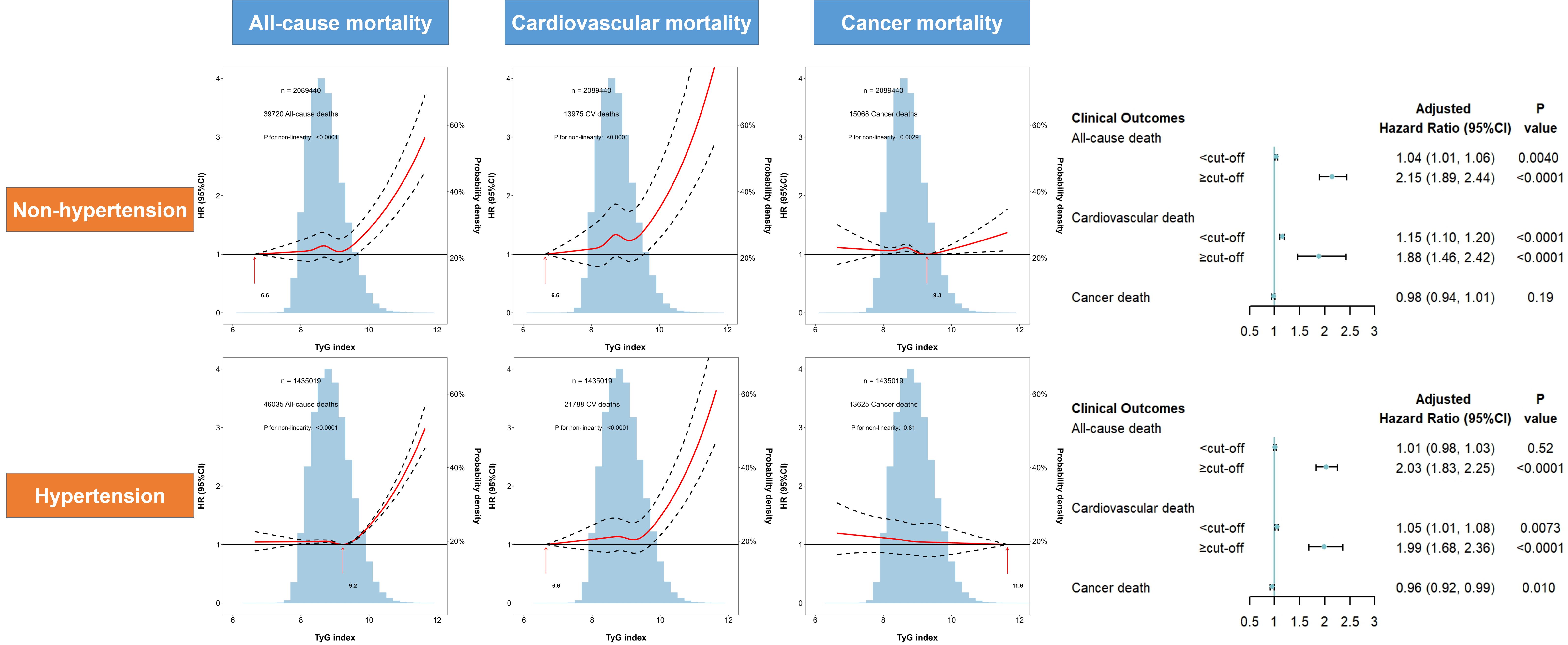
**

**Figure S7. Adjusted association between triglyceride-glucose index and mortality by diabetes.**

**
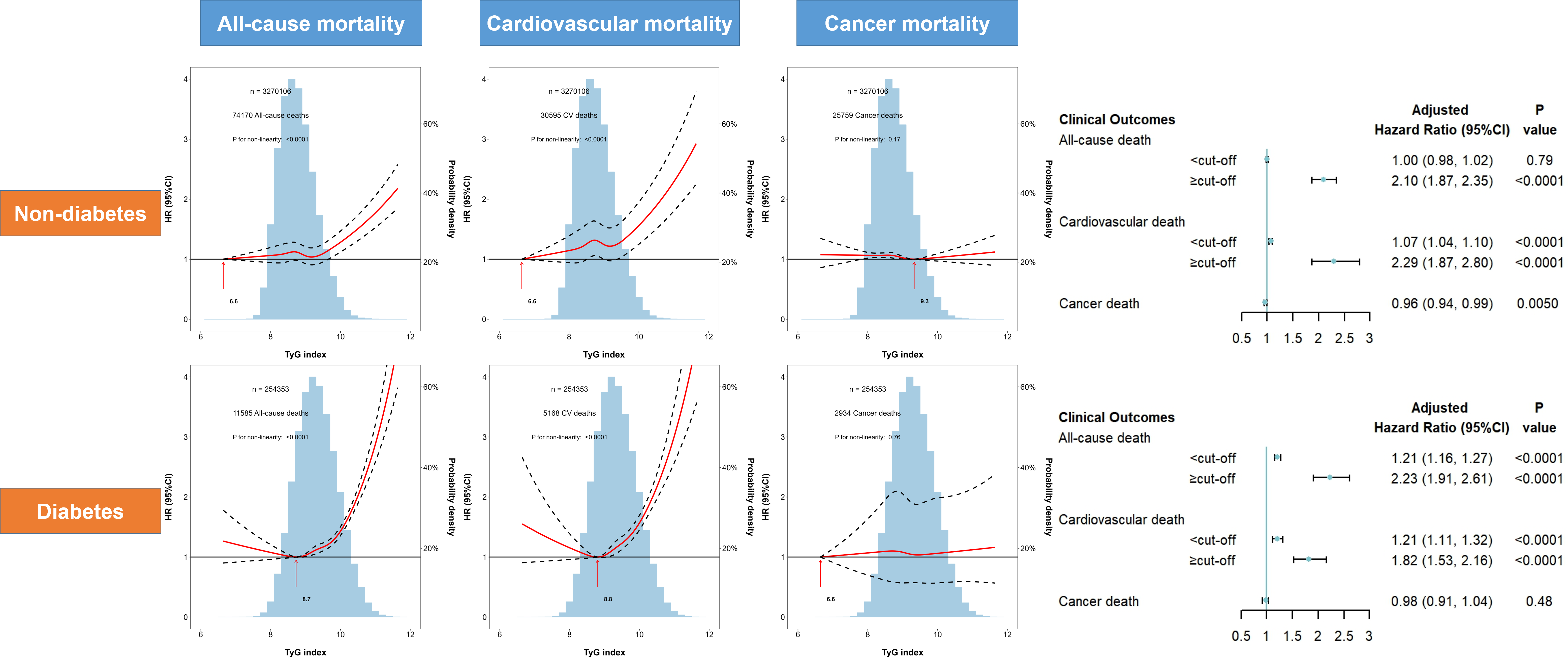
**

**Figure S8. Adjusted association between triglyceride-glucose index and mortality by antidiabetic drugs among participants with diabetes.**

**
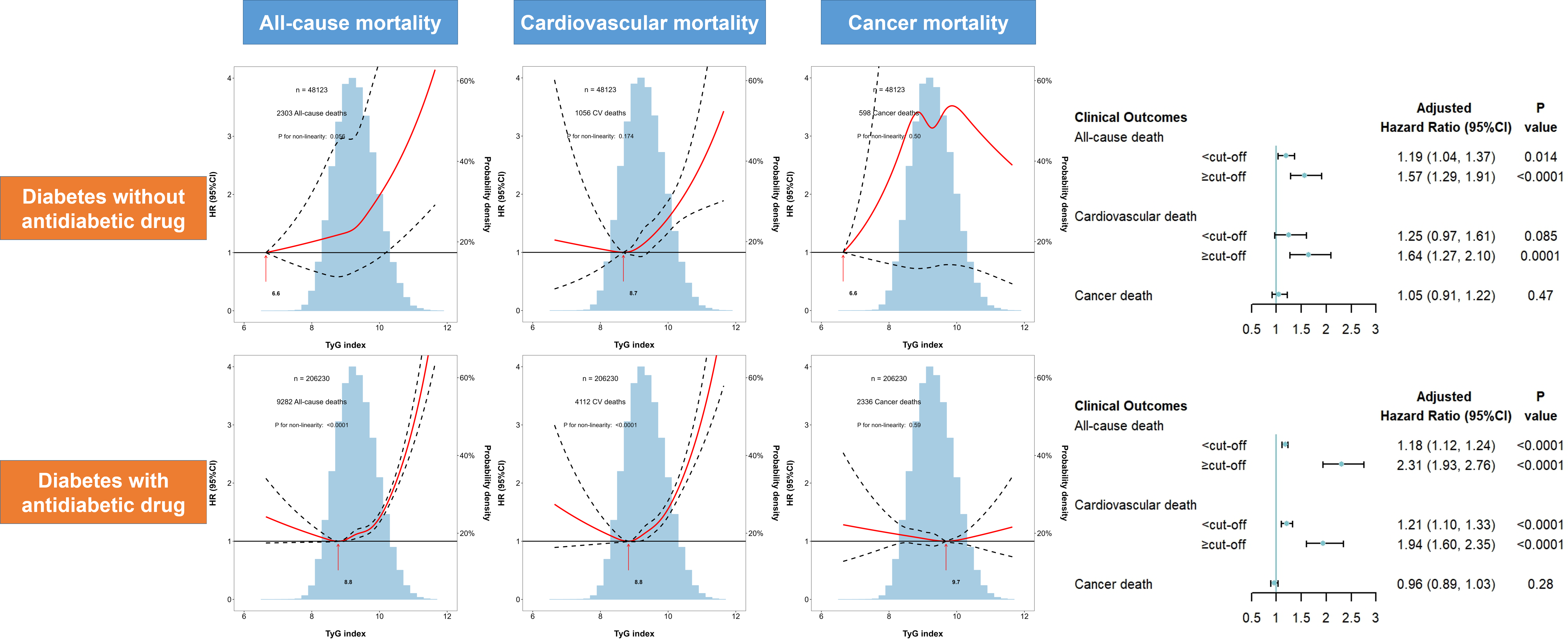
**

**Figure S9. Sensitivity analyses of adjusting for non-high-density lipoprotein cholesterol.**

**
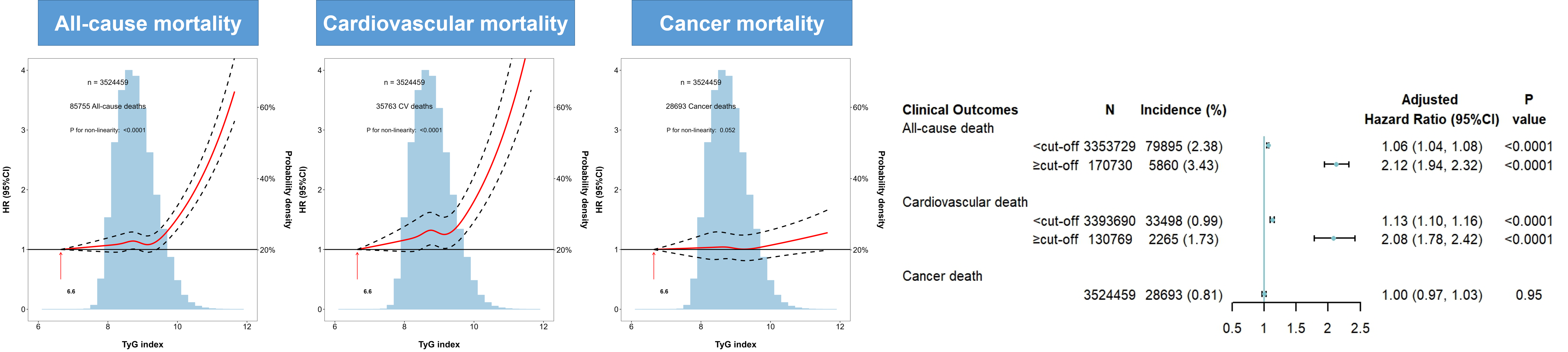
**

**Figure S10. Sensitivity analyses among participants without using lipid-lowering drugs.**

**
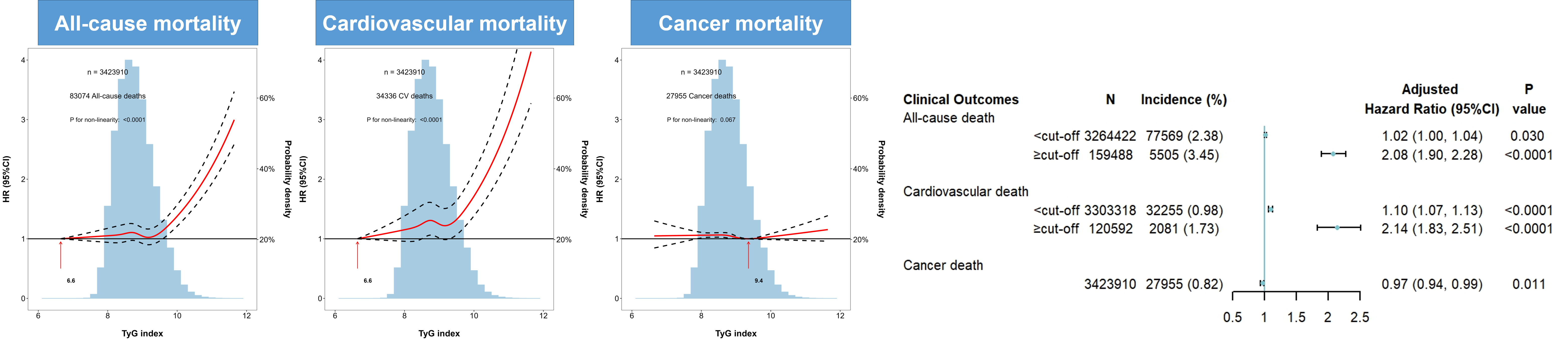
**
